# Penicamide A, A Unique *N*,*N*′-Ketal Quinazolinone Alkaloid from Ascidian-Derived Fungus *Penicillium* sp. 4829

**DOI:** 10.3390/md17090522

**Published:** 2019-09-05

**Authors:** Senhua Chen, Minghua Jiang, Bin Chen, Jintana Salaenoi, Shah-Iram Niaz, Jianguo He, Lan Liu

**Affiliations:** 1 School of Marine Sciences, Sun Yat-sen University, Guangzhou 510006, China (S.C.) (M.J.) (B.C.) (J.H.); 2 Southern Laboratory of Ocean Science and Engineering (Guangdong, Zhuhai), Zhuhai 519000, China; 3 Department of Marine Science, Faculty of Fisheries, Kasetsart University, Bangkok 10900, Thailand; 4 Institute of chemical sciences, Gomal University, Dera Ismail Khan 27100, Pakistan

**Keywords:** ascidian-derived fungus, quinazolinone alkaloid, *Penicillium*, anti-inflammatory

## Abstract

Previously unreported *N*,*N*′-ketal quinazolinone enantiomers [(−)-**1** and (+)-**1**] and a new biogenetically related compound (**2**), along with six known compounds, 2-pyrovoylaminobenzamide (**3**), *N*-(2-hydroxypropanoyl)-2 amino benzoic acid amide (**4**), pseurotin A (**5**), niacinamide (**6**), citreohybridonol (**7**), citreohybridone C (**8**) were isolated from the ascidian-derived fungus *Penicillium* sp. 4829 in wheat solid-substrate medium culture. Their structures were elucidated by a combination of spectroscopic analyses (1D and 2D NMR and Electron Circular Dichroism data) and X-ray crystallography. The enantiomeric pair of **1** is the first example of naturally occurring *N*,*N*′-ketal quinazolinone possessing a unique tetracyclic system having 4-quinazolinone fused with tetrahydroisoquinoline moiety. The enantiomeric mixtures of **1** displayed an inhibitory effect on NO production in lipopolysaccharide-activated RAW264.7 cells, while the optically pure (–)-**1** showed better inhibitory effect than (+)-**1**.

## 1. Introduction

Ascidians have continued to attract a widespread interest for a long time as an important resource for marine natural products with novel structures and potent pharmacological activities [1,2]. One of the most famous marine natural products is the anticancer drug trabectedin (ET-743), which was initially isolated from the marine ascidian *Ecteinascidia turbinata* [3,4]. More and more research suggests that the real producer of ET-743 is the endosymbiont *γ*-proteobacterium *Candidatus Endoecteinascidia frumentensis* [5]. Currently, more scientists have paid attention to ascidian-derived microorganisms, from which more than 150 secondary metabolites with promising bioactivities have been discovered up to now [6]. For example, novel antitumor antibiotics lomaiviticins A and B containing unique dimeric diazobenzofluorene glycoside structures were obtained from ascidian-associated actinomycetes *Micromonospora lomaivitiensis* [7]; the antimycobacterial oxazinin A, with an intricate dimeric structure, was discovered from a new strain of filamentous ascidian-associated fungus, *Eurotiomycetes* strain 110162 [8].

**Figure 1:**
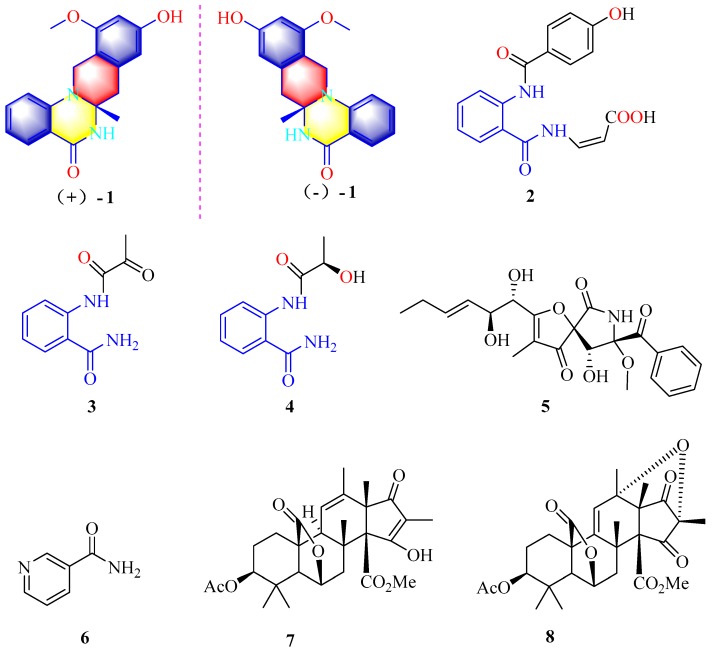
Chemical structures of **1**–**8**.

Our research group has focused on the secondary metabolites of ascidian-derived fungi isolated from the South China Sea in recent years [9,10]. The ascidian-derived fungus *Penicillium* sp. 4829 was isolated from the ascidian *Styela plicata*, collected from the South China Sea. Subsequent chemical investigation led to the isolation of a pair of novel *N*,*N*′-ketal quinazolinone enantiomers, (±)-penicamide A [(−)-**1** and (+)-**1**] and one new biogenetically related compound, penicamide B (**2**), along with six known compounds, 2-pyruvamidobenzamide (**3**) [11], (*S*)-2-(2-hydroxypropanamido)benzamide (**4**) [12], pseurotin A(**5**) [13], niacinamide (**6**) [14], citreohybridonol (**7**) [15], and citreohybriddione C (**8**) [16] (Figure 1). (±)-Penicamide A (**1**) is the first example of naturally occurring *N*,*N*′-ketal quinazolinone possessing a unique tetracyclic system having 4-quinazolinone fused with tetrahydroisoquinoline. Herein, details of the isolation, structural elucidation, hypothetical biogenetic pathway, as well as biological activity of compounds **1**−**8** are reported.

## 2. Results and Discussion

Penicamide A (**1**) was obtained as a white solid. The molecular formula was determined as C_18_H_18_N_2_O_3_ on the basis of the positive HR-ESIMS (High Resolution Mass Spectrometry) (Figure S1) ions at *m*/*z* 311.1389 [M + H]^+^ (calcd. for 311.1390, C_18_H_19_N_2_O_3_), implying 11 degrees of unsaturation. The ^1^H NMR spectrum (Figure S2) (Table 1) along with HSQC experiment (Figure S4) showed resonances for an amide proton [*δ*_H_ 8.42 (1H, s)], a phenolic hydroxyl group [*δ*_H_ 9.40 (1H, s)], six aromatic protons owing to a 1,2-disubstituted aromatic ring [*δ*_H_ 7.75 (1H, dd, *J* = 1.6, 7.7 Hz); 6.85 (1H, m); 7.45 (1H, t, *J* = 8.5 Hz); 6.85 (1H, m)] and a 1,2,3,5-tetrasubstituted aromatic ring [*δ*_H_ 6.32 (1H, d, *J* = 1.6 Hz); 6.16 (1H, d, *J* = 1.6 Hz)], two methylenes with AB coupling system [*δ*_H_ 3.76 (1H, d, *J* = 16.4 Hz) and 4.45 (1H, d, *J* = 16.4 Hz); 2.80 (1H, d, *J* = 15.9 Hz) and 3.08 (1H, d, *J* = 15.9 Hz)], one methoxy [*δ*_H_ 3.80 (3H, s)], and one methyl [*δ*_H_ 1.20 (3H, s)]. The ^13^C NMR (Figure S3) and HSQC data (Table 1) of **1** revealed the presence of 18 carbons for 13 sp^2^ hybridized carbons and five sp^3^ hybridized carbons including one carbon (*δ*_C_ 68.9) connected with heteroatoms.

**Figure 2:**
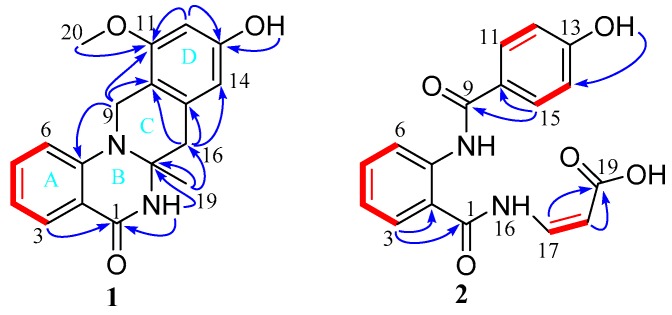
Key ^1^H–^1^H COSY (red line) and HMBC (blue arrow) correlations of compounds **1** and **2**.

A 4-quinazolinone moiety (rings A and B) was assigned by ^1^H–^1^H COSY correlations (Figure S5) between H-3 and H-4, H-4 and H-5, H-5 and H-6, and the HMBC correlations (Figure S6) from aromatic proton H-3 to C-1 and C-2, H-6 to C-2 and C-7, and the amide proton 18-NH to C-1 and C-17, as well as their chemical shift (Figure 2). The HMBC correlations of H-12 with C-11 and C-13, H-14 with C-10 and C-15, 13-OH with C-13, and H-20 with C-11 established a 1,2,3,5-tetrasubstituted benzene group. The key HMBC correlations from H-9 to C-7, C-10, and C-11, H-16 to C-10, C-14, C-15, and C-17, together with the chemical shift (*δ*_C_ 42.1, C-9; 41.7, C-16; 68.9, C-17) suggested that the phenyl group (ring D) was connected to the *N*-8 of 4-quinazolinone moiety through methylene C-9. At the same time, HMBC correlations from H-16 to C-10, C-14, C-15, and C-17 indicated that C-15 of phenyl group was linked to C-17 of 4-quinazolinone moiety via methylene C-16 to form the C ring. The remaining methyl group was located at C-17, supported by the HMBC correlation of H_3_-19 with C-16 and C-17.

**Figure 3:**
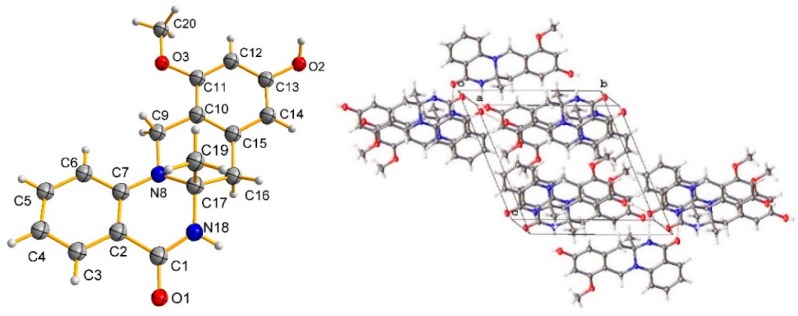
Single crystal structure and molecular packing properties of **1**.

**Figure 4:**
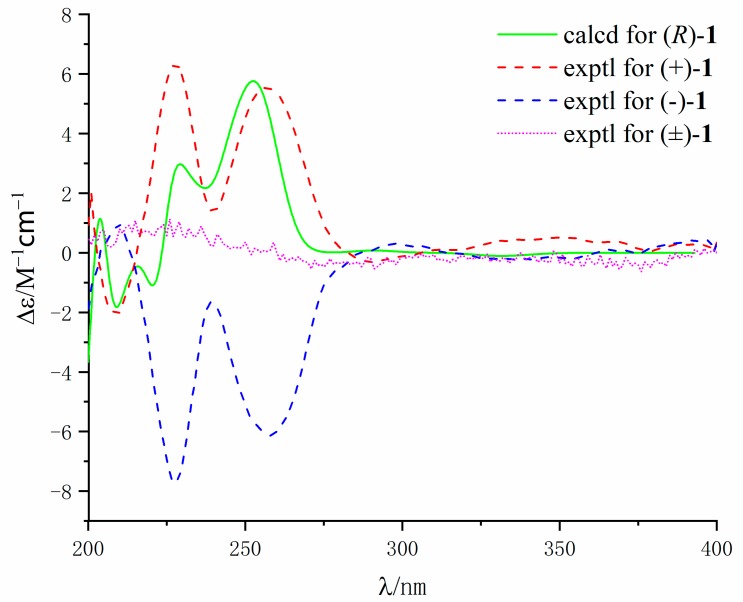
Experimental CD spectra of (−)-**1**, (+)-**1,** and (±)-**1** in MeOH and the calculated ECD spectrum of (*R*)-**1**.

Compound **1** was crystallized from methanol by slow evaporation method to afford a crystal of the triclinic space group P^−1^, so the structure of **1** was further confirmed by X-ray crystallography (Figure 3). The P^−1^ space group of the X-ray structure suggested that compound **1** should be an enantiomer in the solid crystals (Figure 3), which agreed with the result of no signal in the CD spectrum and no optical activity sign in methanol. Subsequently, (±)-**1** was subjected to chiral HPLC for purification, and the two enantiomers, (+)-**1** (*t*_R_ = 28.7 min) and (−)-**1** (*t*_R_ = 31.5 min) were obtained, respectively. They displayed opposite Cotton effects in their CD spectra and opposite optical rotations (Figure 4). The calculated ECD data of (*R*)-**1** were comparable to the experimental one of (+)-**1**. This result suggested that the absolute configuration of (+)-**1** was *R* and (−)-**1** was *S*. Thus, (+)-**1** and (–)-**1** was named as (+)-penicamide A and (−)-penicamide A, respectively.

Penicamide B (**2**) was isolated as a white powder. The molecular formula was determined as C_17_H_14_O_5_N_2_ according to its HR-ESIMS (Figure S9) (*m*/*z* 325.0835 [M − H]^−^, calcd. for C_17_H_13_O_5_N_2_, 325.0830). The ^1^H NMR data (Table 1) (Figure S11) revealed the presence of eight aromatic protons [*δ*_H_ 7.99 (1H, d, *J* = 8.3 Hz); 7.19 (1H, t, *J* = 7.4 Hz); 7.62 (1H, m); 8.47 (1H, d, *J* = 8.3 Hz); 7.76 (2H, d, *J* = 8.7 Hz); 6.95 (2H, d, *J* = 8.7 Hz)] and two olefinic protons [*δ*_H_ 7.64 (1H, m); 5.46 (1H, d, *J* = 8.7 Hz)]. In the ^13^C NMR spectrum (Figure S12), seventeen carbon signals all appeared in the downfield shift and belonged to sp^2^ hybridized carbons, seven of which were attributed to unprotonated carbons (*δ*_H_ 169.4, 117.8, 140.0, 163.0, 122.4, 161.9, 167.4).

2-Aminobenzamide moiety was established by the HMBC (Figure S15) correlations of H-3 with C-1 and C-2, H-6 with C-2 and C-7, as well as the ^1^H–^1^H COSY (Figure S14) correlations of H-3/H-4/H-5/H-6 (Figure 3). The chemical shift and coupling constants of NH-16 (*δ*_H_ 12.09, d, *J* = 10.6 Hz), CH-17 (*δ*_H_ 7.64, m) and CH-8 (*δ*_H_ 5.46, d, *J* = 8.7 Hz) enabled the connection of the C-8 with C-17 of a Z geometry acrylic acid group, which was identified by the HMBC correlations from H-17 and H-18 to C-19, and the ^1^H–^1^H COSY of H-17 with H-18. The HMBC correlations from 13-OH to C-12 and C-14, from H-11 and H-15 to C-9 and C-10, together with the ^1^H–^1^H COSY correlations of H-12/H-13 and H-14/H-15 revealed the presence of 4-hydroxybenzamide unit, which was linked to NH-8 of 2-aminobenzamide moiety according to the chemical shift of C-9 (*δ*_C_ 163.0).

**Figure 5:**
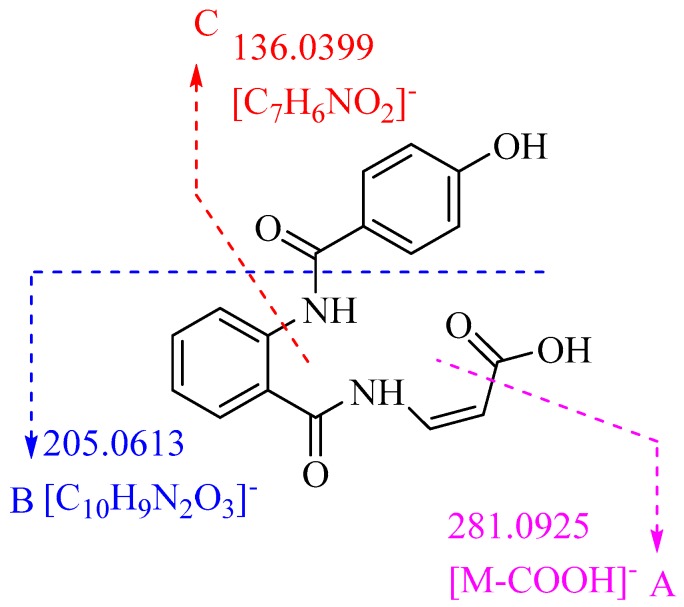
Identified sequences for ESI-HRMS/MS fragments of **2**.

The residue sequence of **2** was confirmed by high resolution ESI-HRMS/MS (High Resolution Mass Spectrometry) analysis (Figure S10). Figure 5 shows the assignment of observed secondary ions of the negative [M − H]^−^ ion (*m*/*z* 325.0835) that were unambiguously assigned by high resolution mass determination. Fragment A confirmed the sequence which the molecule was lost of a carboxyl residue, while fragment C was fully consistent with the 4-hydroxybenzamide unit. Fragment B was also well agreement with the sequence [M − C_7_H_6_O_2_]^−^. Hence, the structure of **2** was elucidated as (*Z*)-3-(2-(4-hydroxybenzamido)benzamido)acrylic acid and named as penicamide B.

**Figure 6:**
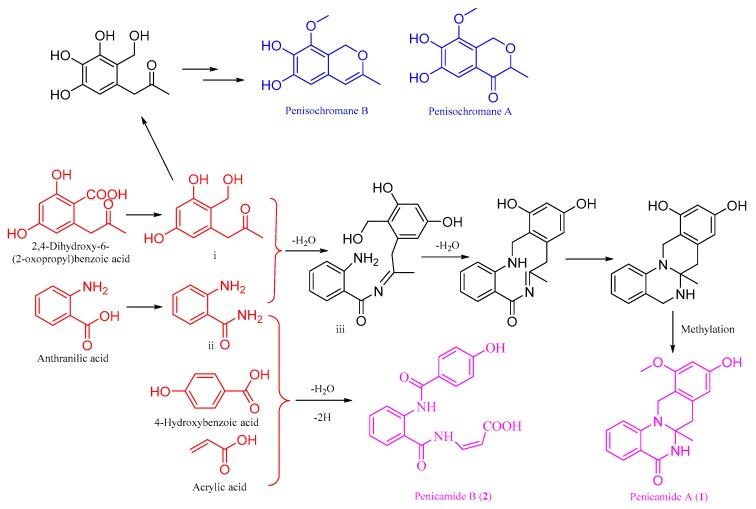
Plausible biogenetic pathway leading to a formation of (±)-penicamide A.

Structurally, (±)-penicamide A is the first example of *N*,*N*′-ketal quinazolinone alkaloid that possesses a unique tetracyclic system 6/6/6/6 with 4-quinazolinone fusing with tetrahydroisoquinoline. On the one hand, some phenylpropanoid derivatives, penisochromanes A and B have been isolated from *Penicillium* sp. 4829 previously [10]. On the other hand, two known anthranilic acid derivatives, 2-pyrovoylaminobenzamide (**3**) and *N*-(2-hydroxypropanoyl)-2 amino benzoic acid amide (**4**) were isolated from *Penicillium* sp. 4829 together herein. So, (±)-penicamide A (**1**) should be PKS–NRPS hybrid metabolites derived from anthranilic acid and phenylpropanoid, 2,4-dihydroxy-6-(2-oxopropyl)benzoic acid. A possible biogenetic pathway for **1** was proposed as shown in Figure 6. Two intermediates i and ii were derived from the precursors anthranilic acid and 2,4-dihydroxy-6-(2-oxopropyl)benzoic acid, followed by dehydration–condensation to generate intermediate Schiff base iii. Afterward, iii underwent dehydration, cyclization, and methylation to give (±)-penicamide A with *N*,*N*′-ketal quinazolinone skeleton. Penicamide B (**2**) might be derived from 4-hydroxybenzoic acid, acrylic acid, and intermediate ii with dehydration–condensation and dehydrogenation.

Quinazoline alkaloids have long been explored as invaluable sources of inspiration for drug discovery due to their diverse pharmacological activities [17,18,19], such as antifungal [20], antitubecular [21], antibacterial [22], antiviral [23], antimalarial [24], anti-inflammatory [25], and antitumor [26]. Compounds **1**–**8** were tested for their anti-inflammatory activity against LPS-activated NO production in RAW264.7 cells in vitro using the Griess assay with indomethacin as a positive control (IC_50_ = 36.3 ± 1.5 µM). The enantiomer mixture (±)-penicamide A (1) displayed moderate inhibitory effect on NO production with IC_50_ values of 35.1 ± 1.7 µM, while the optically pure (–)-**1** showed better inhibitory effect than (+)-**1** [IC_50_: 27.2 ± 1.2 µM for (–)-**1** and 47.5 ± 2.3 µM for (+)-**1**]. In addition, **2** and **4** also exhibited moderate anti-inflammatory activity with IC_50_ values of 45.9 ± 2.0 and 21.8 ± 1.3 µM, respectively. The other compounds displayed weak or no anti-inflammatory activity at a concentration of 50 µM. All the isolated compounds were evaluated for their cytotoxicity against A549 (lung cancer), HeLa (cervical cancer), and MCF-7 (breast cancer) human cancer cell lines using MTT assay and displayed no cytotoxicity against all the three cell lines at a concentration of 50 µM.

## 3. Materials and Methods

### 3.1. General Experimental Procedures

Optical rotations were measured using an MCP 200 (Anton Paar, Shanghai, China) polarimeter. UV spectra were recorded on a Shimadzu UV-240 spectrophotometer (Shimadzu, Kyoto, Japan). The Chirascan-plus circular dichroism spectrometer (Applied Photophysics Ltd., Surrey, UK) was used for recording ECD spectra. IR spectra were recorded by a Fourier transformation infra-red spectrometer coupled with infra-red microscope EQUINOX 55 (Bruker, Rheinstetten, Germany). The 1D and 2D NMR spectra were recorded on Bruker Avance 400 spectrometer (^1^H 400 MHz, ^13^C 100 MHz). Chemical shifts (*δ*) were given in ppm with reference to the solvent signal (*δ*_C_ 39.52/*δ*_H_ 2.50 for DMSO), and coupling constants (*J*) were given in Hz. HR-ESIMS spectra were recorded on an LTQ-Orbitrap LC-MS spectrometer (Thermo Corporation, Waltham, MA, USA). ESIMS spectra were obtained on an ACQUITY QDA LC-MS spectrometer (Waters Corporation, Milford, MA, USA). Column chromatography (CC) was performed using silica gel (200–300 mesh, Qingdao Marine Chemical Factory, China) and Sephadex LH-20 (GE Healthcare, Littile Chalfont, UK) as stationary phases. HPLC was performed on an Essentia LC-16 (Shimadzu, Shanghai, China). Pre-coated silica gel plates (Qingdao Huang Hai Chemical Group Co., G60, F-254, China) were used for thin layer chromatography. 

### 3.2. Biological Material

The strain was isolated from the ascidian *Styela plicata*, which was collected in the Bay of Da’ao, Shenzhen City, Guangdong, Province, China, in April 2016. The fungus was obtained using the standard protocol for isolation [27]. Fungal identification was carried out using a molecular biological protocol by DNA amplification and sequencing of the ITS (Internal Transcribed Spacer) region. The sequence data obtained from the fungal strain have been deposited at GenBank with an accession no. MH465534. A BLAST search result showed that the sequence was most similar (99%) to the sequence of *Penicillium canescens* (compared to KJ027992.1). A strain was deposited in the Guangdong Microbial Culture Center (GDMCC) under the patent depository number GDMCC No. 60402.

### 3.3. Extraction, Isolation, and Characterization

The fungus was cultured on a wheat solid-substrate medium (sixty 500 mL Erlenmeyer flasks; each containing 50 g of wheat, 1.5 g of artificial sea salt, and 50 mL of distilled H_2_O) at room temperature under static conditions and daylight for four weeks. Following incubation, the mycelia and solid wheat media were extracted with EtOAc three times and the EtOAc solutions was combined and evaporated under reduced pressure to give 32.4 g of a crude EtOAc extract, which was chromatographed on a silica gel (400 g) column and eluted with a gradient of petroleum ether/EtOAc from 80:20 to 0:100 to give seven fractions (Fr. 1–Fr. 7). Fr. 4 (832.1 mg) was subsequently applied on Sephadex LH-20 CC eluting with CH_2_Cl_2_/MeOH (*v*/*v*, 1:1) to give six subfractions. Fr. 4.3 (100.9 mg) was applied on a silica gel (25 g) column and eluted with CH_2_Cl_2_/MeOH *v*/*v*, 96:4 to give **7** (6.1 mg) and **8** (8.9 mg), respectively. Fr. 5 (1.32 g) was subsequently applied on a Sephadex LH-20 (50 g) column and eluted with CH_2_Cl_2_/MeOH *v*/*v*, 1:1 to give subfraction Fr. 5.6, which was chromatographed on a silica gel (25 g) column and eluted with CH_2_Cl_2_/MeOH *v*/*v*, 97:3 to yield **1** (2.8 mg). The enantiomeric mixture of **1** (2.2 mg) was applied on a chiral HPLC (90% n-hexane/isopropyl alcohol, flow rate 1 mL/min, Ultimate Cellu-D column 4.6 × 250 mm, 5 µm) to yield (+)-1 (*t*_R_ = 28.7 min, 1.0 mg) and (−)-**1** (*t*_R_ = 31.5 min, 1.1 mg). Fr. 6 (919.5 mg) was subsequently applied on a Sephadex LH-20 CC eluting with CH_2_Cl_2_/MeOH *v*/*v*, 1:1 to give five subfractions. Fr. 6.3 (15.1 mg) was purified by a silica gel (20 g) column and eluted with CH_2_Cl_2_/MeOH *v*/*v*, 95:5 to afford **2** (2.4 mg). Fr. 6.4 (120.4mg) was purified by silica gel (25 g) column) and eluted with CH_2_Cl_2_/MeOH *v*/*v*, 96:4) to afford **3** (5.1 mg) and **4** (7.3 mg). Fr. 7 (679.9 mg) was subsequently subjected to Sephadex LH-20 (50 g) column and eluted with CH_2_Cl_2_/MeOH *v*/*v*, 1:1 to give six subfractions. Fr. 7.3 (89.3 mg) was then purified on silica gel (25 g) column and eluted with CH_2_Cl_2_/MeOH *v*/*v*, 96:4 to afford **5** (10.8 mg) and **6** (5.2 mg).

#### 3.3.1. Penicamide A (**1**)

White solid; mp 174–176 °C; UV (MeOH) *λ*_max_ (log ε) 225 (4.12), 266 (3.78), 349 (3.23) nm; IR (neat) *ν*_max_: 3234, 2929, 1658, 1610, 1488, 1290, 1150, 1108, and 755 cm^−1^; ^1^H and ^13^C NMR spectroscopic data, see Table 1; HR-ESI-MS (High Resolution Mass Spectrometry) 309.1227 [M + H]^+^ (calcd. for 309.1233, C_18_H_17_N_2_O_3_).

(+)-(**1**): ${\left[\alpha \right]}_{D}^{20}$ + 28.5 (c 0.02, MeOH); ECD (MeOH) λ_max_ (Δε): 209 (−1.8), 228 (+6.3), 257 (+5.6) nm.
(−)-(**1**): ${\left[\alpha \right]}_{D}^{20}$ − 30.1 (c 0.02, MeOH); ECD (MeOH) *λ*_max_ (Δε): 210 (+1.0), 227 (−7.7), 257 (−6.1) nm.

#### 3.3.2. Penicamide B (**2**)

White solid; mp 181–183 °C; UV (MeOH) *λ*_max_ (log ε) 322 (3.96) nm; IR (neat) *ν*_max_: 3406, 3336, 3227, 2919, 2850, 1680, 1618, 1584, 1447, 1270, 1195, 848, 786, and 752 cm^−1^; ^1^H and ^13^C NMR spectroscopic data, see Table 1; HR-ESI-MS (*m*/*z* 325.0835 [M − H]^−^, calcd. for C_17_H_13_O_5_N_2_, 325.0830).

### 3.4. X-ray Crystallographic Analysis of Compound 1

Compound **1** was acquired as colorless crystals from CH_3_OH using the vapor diffusion method. The single crystal X-ray diffraction data was obtained on a Rigaku Oxford Diffraction with Cu-Kα radiation (*λ* = 1.54178 A). The structures were solved by direct methods using the SHELXS-97 program (University of Göttingen, Göttingen, Germany) and refined using full-matrix least-squares difference Fourier techniques using Olex2 software (Durham University, Durham, UK). Crystallographic data for **1** have been deposited with the Cambridge Crystallographic Data Centre. Copies of the data can be obtained, free of charge, on application to the Director, CCDC, 12 Union Road, Cambridge CB2 1EZ, UK (fax: 44-(0)1223-336033, or e-mail: deposit@ccdc.cam.ac.uk).

Crystal data of (**1**): C_18_H_18_N_2_O_3_, Mr = 310.34, triclinic, *a* = 5.9198(6) Å, *b* = 11.5313(9) Å, *c* = 11.6390(8) Å, *α* = 66.749(7), *β* = 88.243(7), *γ* = 79.762(7), *V* = 717.68(10) Å^3^, space group P^−1^, *Z* = 2, *D*_calcd_ = 1.436 mg/m^3^, *μ* = 0.805 mm^−1^, and *F*(000) = 328.0. Crystal dimensions: 0.08 × 0.05 × 0.02 mm^3^. Independent reflections: 2838 (*R*_int_ = 0.0448). The final *R*_1_ values were 0.0474, *ωR*_2_ = 0.1324 (*I* > 2*σ*(I)). The goodness of fit on *F*^2^ was 1.031. CCDC number: 1820872.

### 3.5. Calculation of the ECD Spectra

Molecular Merck force field (MMFF) and DFT/TD-DFT calculations were carried out with Spartan 14 software (Wavefunction Inc., Irvine, CA, USA) and Gaussian 09 program, respectively [28]. Conformers within 10 kcal/mol energy window were generated and optimized using DFT calculations at the B3LYP/6-31G(d) level. Conformers with Boltzmann distribution over 1% were chosen for ECD calculations in methanol at the B3LYP/6-311+g(2d,p) level. The IEF-PCM solvent model for MeOH was used. ECD spectra were generated using the program SpecDis 3.0 (University of Würzburg, Würzburg, Germany) and OriginPro 8.5 (OriginLab, Ltd., Northampton, MA, USA) from dipole-length rotational strengths by applying Gaussian band shapes with sigma = 0.30 eV. All calculations were performed by Tianhe-2 in the National Supercomputer Center in Guangzhou.

### 3.6. Cytotoxic Assay

All the isolated compounds were evaluated for their cytotoxicity against three human cancer cell lines, A549 (lung cancer), HepG2 (liver cancer), and MCF-7 (breast cancer). The three tumor cell lines were generously provided by the cell bank of the Chinese Academy of Sciences (Shanghai, China). The cytotoxic activity of the tested compounds were assayed by the MTT method using 96 well plates according to the previously reported procedure [29]. 

### 3.7. Anti-Inflammation Bioassays

The anti-inflammatory activity of the isolated compounds was evaluated according to the previously reported method [30].

## 4. Conclusions

More and more attention has been focused on ascidian-derived microorganisms, from which more than 150 potential bioactive secondary metabolites have been discovered up to date. A pair of novel *N*,*N*′-ketal quinazolinone enantiomers [(−)-**1** and (+)-**1**] and one new biogenetically related compound (**2**) were isolated from ascidian-derived fungus *Penicillium* sp. 4829 in wheat solid-substrate medium culture. (±)-Penicamide A (**1**) was the first example of natural *N*,*N*′-ketal quinazolinone enantiomers possessing a unique tetracyclic system 6/6/6/6 with 4-quinazolinone fusing with tetrahydroisoquinoline. (±)-penicamide A (**1**) should be PKS–NRPS hybrid metabolites derived from anthranilic acid and 2,4-dihydroxy-6-(2-oxopropyl)benzoic acid. The enantiomeric mixture of (±)-penicamide A (**1**) displayed potential inhibitory effects on NO production in lipopolysaccharide activated in RAW264.7 cells, while the optically pure (–)-**1** showed better inhibitory effects than that of (+)-**1**. 

## Figures and Tables

**Table 1:** ^1^H (400 MHz) and ^13^C (100 MHz) NMR data of **1** and **2** in DMSO-*d*_6_.

No.	1		2	
	*δ*_C_, Type	*δ*_H_, (*J* in Hz)	*δ*_C_, Type	*δ*_H_, (*J* in Hz)
1	161.8, C		169.4, C	
2	116.3, C		117.8, C	
3	127.4, CH	7.75, dd (1.6, 7.7)	131.2, CH	7.99, d (8.3)
4	117.9, CH	6.85, m	123.5, CH	7.19, t (7.4)
5	133.9, CH	7.45, t (8.5)	133.8, CH	7.62, m
6	112.5, CH	6.85, m	127.4, CH	8.47, d (8.3)
7	147.1, C		140.0, C	
8				
9	42.1, CH_2_	3.76, d (16.4) 4.45, d (16.4)	163.0, C	
10	109.9, C		122.4, C	
11	156.6, C		129.5, CH	7.76, d (8.7)
12	97.1, CH	6.32, d (1.6)	115.9, CH	6.95, d (8.7)
13	157.2, C		161.9, C	
14	106.3, CH	6.16, d (1.6)	115.9, CH	6.95, d (8.7)
15	132.8, C		129.5, CH	7.76, d (8.7)
16	41.7, CH_2_	2.80, d (15.9) 3.08, d (15.9)		12.09, d (10.6)
17	68.9, C		137.4, CH	7.64, m
18		8.42, s	100.2, CH	5.46, d (8.7)
19	21.3, CH_3_	1.20, s	167.4, C	
20	55.3, CH_3_	3.80, s		
13-OH		9.40, s		

## Supplementary Materials

The following are available online at https://www.mdpi.com/1660-3397/17/9/522/s1, Figure S1–S26: HR-ESIMS, NMR of the new compounds as well as other supporting data, Table S1. Energy Analysis for the Conformers of (R)-1.
