# Supplementary material for: Penicamide A, A Unique N,N′-Ketal Quinazolinone Alkaloid from Ascidian-Derived Fungus Penicillium sp. 4829
Source: Mar Drugs. 2019 Sep 5;17(9):522. doi: 10.3390/md17090522 (PMC6780914; doi:10.3390/md17090522)
Supplement: Supplementary file 1 [file marinedrugs-17-00522-s001.pdf]

Supporting Information

Penicamide A, a Unique *N,N'*-Ketal Quinazolinone Alkaloids from Ascidian-derived Fungus *Penicillium* sp. 4829

Senhua Chen[1,2], Minghua Jiang[1], Bin Chen[1], Jintana Salaenoi [2], Shah-Iram Niaz [1,4,*], Jianguo He [1], and Lan Liu [1,2,*]

[1] School of Marine Sciences, Sun Yat-Sen University, Guangzhou 510004, China;

[2] Southern Laboratory of Ocean Science and Engineering (Guangdong, Zhuhai), Zhuhai 519000, China;

[3] Department of Marine Science, Faculty of Fisheries, Kasetsart University, Bangkok 10900, Thailand.

[4] Institute of chemical sciences, Gomal University ,Dera Ismail Khan, Pakistan；

Correspondence

To whom correspondence should be addressed.

Tel/Fax: +86-20-84725459. E-mail: shah_iram2000@yahoo.com; cesllan@mail.sysu.edu.cn

Pages		
3	**Figure S1**	The HRESI-MS spectrum of compound **1**.
3	**Figure S2**	The ^{1}H NMR (400MHz) spectrum of compound **1** in DMSO-d_6.
4	**Figure S3**	The ^{13}C NMR (100MHz) spectrum of compound **1** in DMSO-d_6.
4	**Figure S4**	The HSQC spectrum of compound **1** in DMSO-d_6.
5	**Figure S5**	The ^{1}H-^{1}H COSY spectrum of compound **1** in DMSO-d_6.
5	**Figure S6**	The HMBC spectrum of compound **1** in DMSO-d_6.
6	**Figure S7**	The NOESY spectrum of compound **1** in DMSO-d_6.
6	**Figure S8**	The IR spectrum of compound **1**.
7	**Figure S9**	The HRESI-MS spectrum of compound **2**.
7	**Figure S10**	The HRESI-MS/MS spectrum of compound **2**.
8	**Figure S11**	The ^{1}H NMR (400MHz) spectrum of compound **2** in DMSO-d_6.
8	**Figure S12**	The ^{13}C NMR (100MHz) spectrum of compound **2** in DMSO-d_6.
9	**Figure S13**	The HSQC spectrum of compound **2** in DMSO-d_6.
9	**Figure S14**	The ^{1}H-^{1}H COSY spectrum of compound **2** in DMSO-d_6.
10	**Figure S15**	The HMBC spectrum of compound **2** in DMSO-d_6.
10	**Figure S16**	The IR spectrum of compound **2**.
11	**Figure S17**	The ^{1}H NMR (400MHz) spectrum of compound **3** in acetone-d_6.
11	**Figure S18**	The ^{13}C NMR (100MHz) spectrum of compound **3** in acetone-d_6.
12	**Figure S19**	The ^{1}H NMR (400MHz) spectrum of compound **4** in MeOH-d_4.
12	**Figure S20**	The ^{13}C NMR (100MHz) spectrum of compound **4** in MeOH-d_4.
13	**Figure S21**	The ^{1}H NMR (400MHz) spectrum of compound **5** in $CDCl_3$.
13	**Figure S22**	The ^{13}C NMR (100MHz) spectrum of compound **5** in $CDCl_3$.
14	**Figure S23**	The ^{1}H NMR (400MHz) spectrum of compound **6** in MeOH-d_4.
14	**Figure S24**	The ^{13}C NMR (100MHz) spectrum of compound **6** in MeOH-d_4.
15	**Figure S25**	The ^{1}H NMR (400MHz) spectrum of compound **7** in DMSO-d_6.
15	**Figure S26**	The ^{13}C NMR (100MHz) spectrum of compound **7** in DMSO-d_6.
16	**Figure S25**	The ^{1}H NMR (400MHz) spectrum of compound **8** in acetone-d_6.
16	**Figure S26**	The ^{13}C NMR (100MHz) spectrum of compound **8** in acetone-d_6.
17	**Experimental Section**	
17	**Table S1.**	Energy Analysis for the Conformers of (*R*)-**1.**
17	**Figure S17**	B3LYP/6-31G(d) optimized low-energy conformers of (*R*)-**1**.
18		NMR data of known compounds

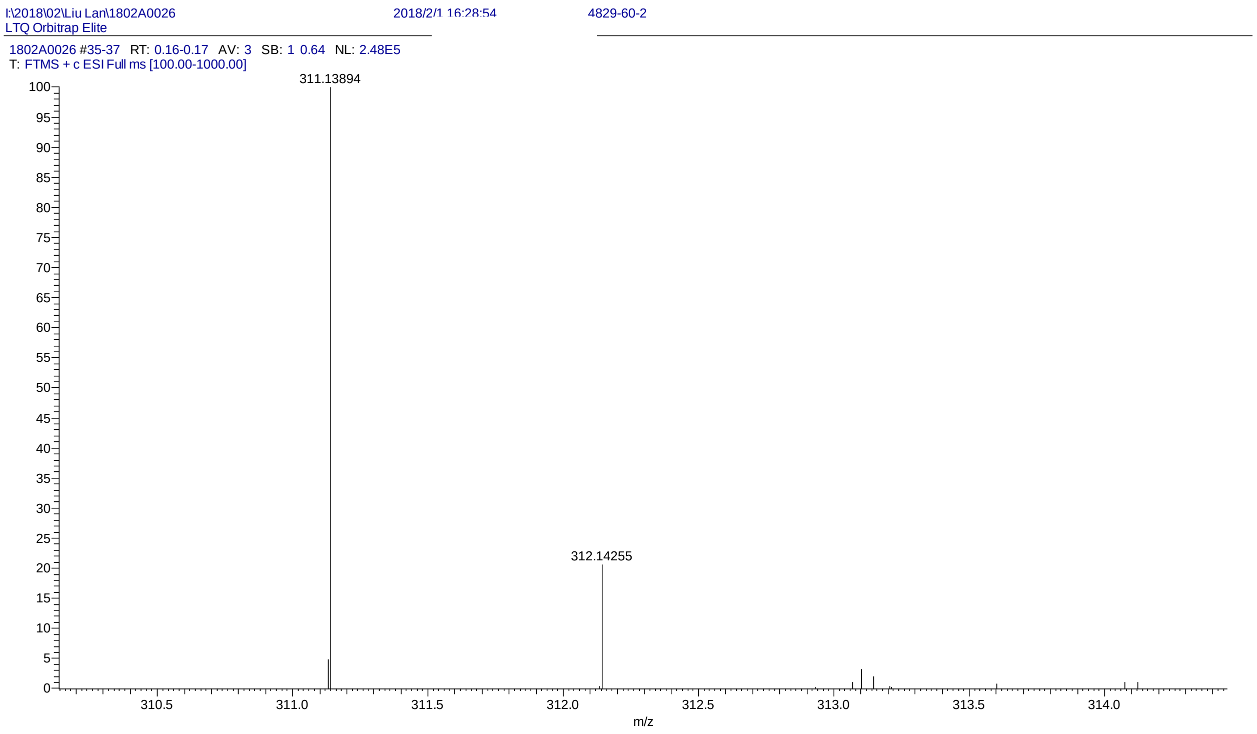

Figure S1. The HRESIMS spectrum of compound **1**.

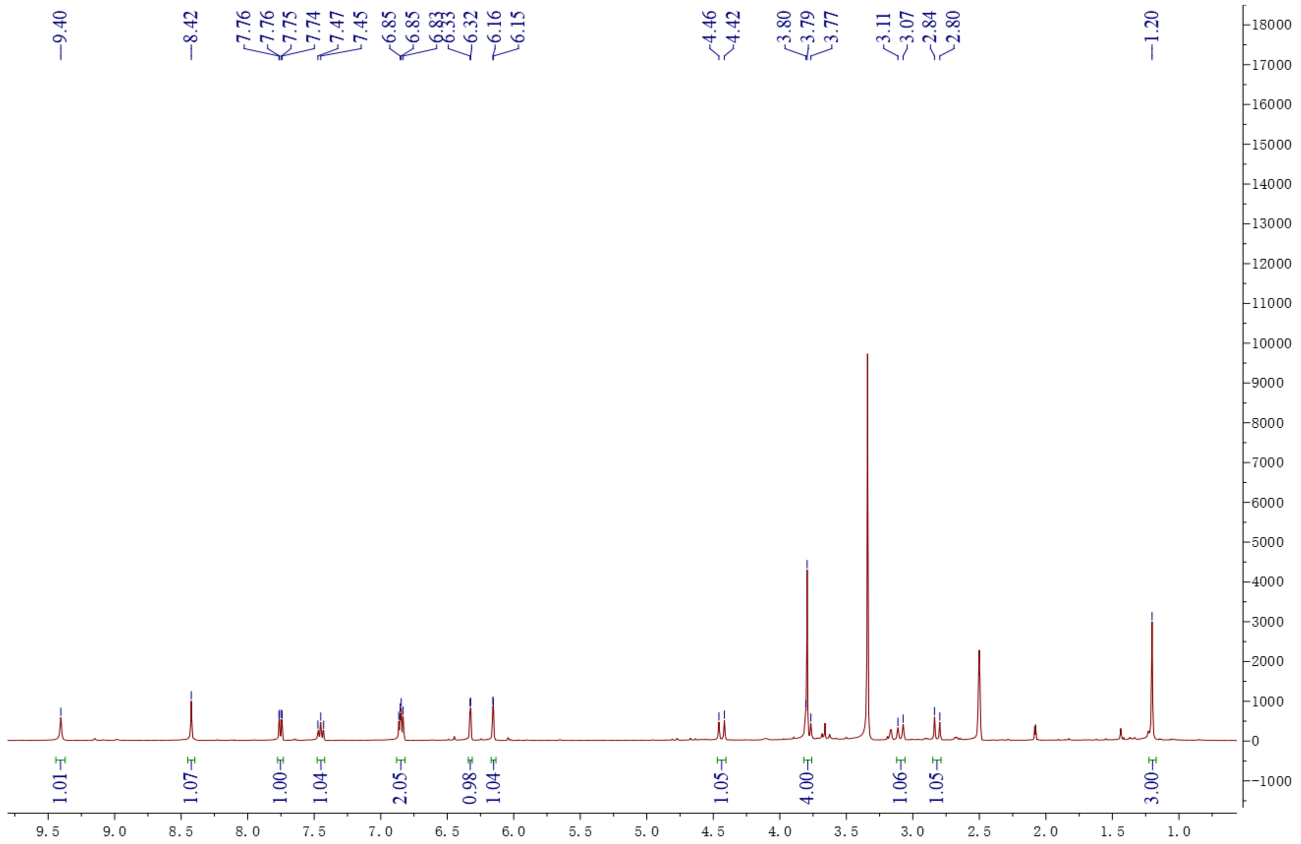

Figure S2. The ^{1}H NMR (400MHz) spectrum of compound **1** in DMSO-d_6.

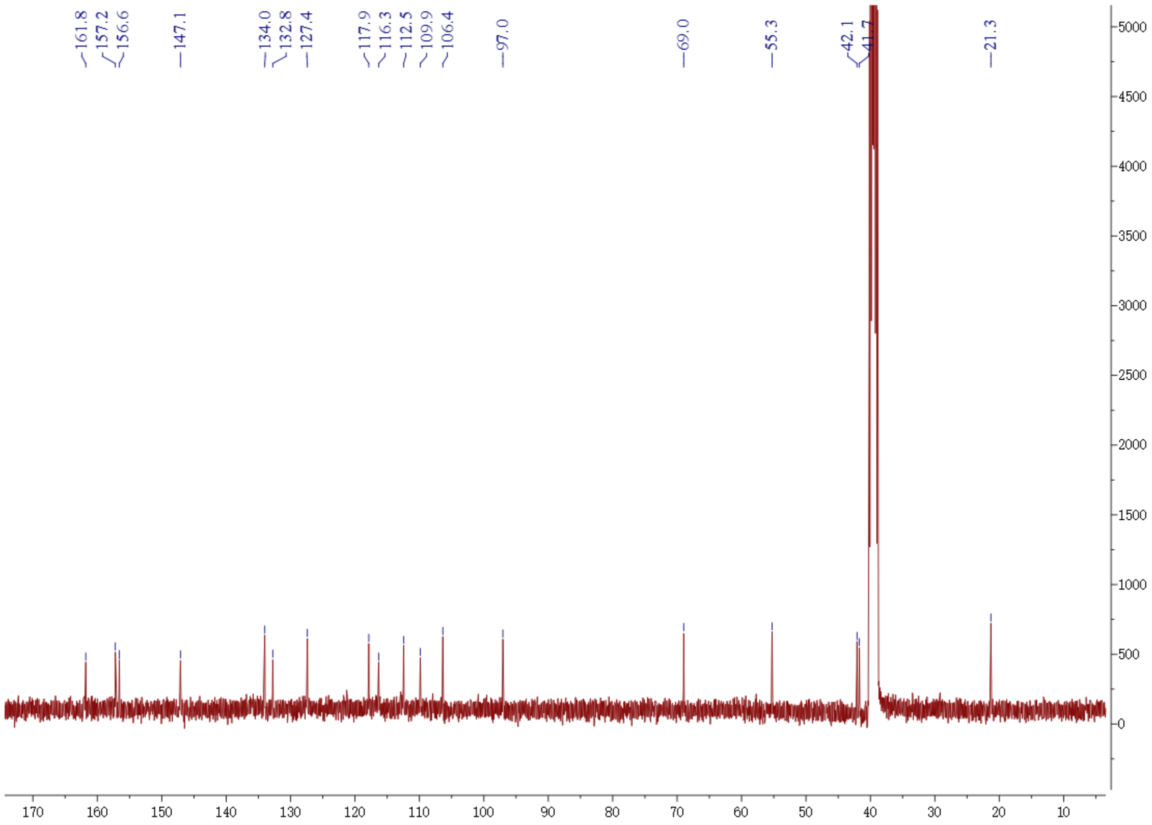

Figure S3. The ^{13}C NMR (100MHz) spectrum of compound **1** in DMSO-d_6.

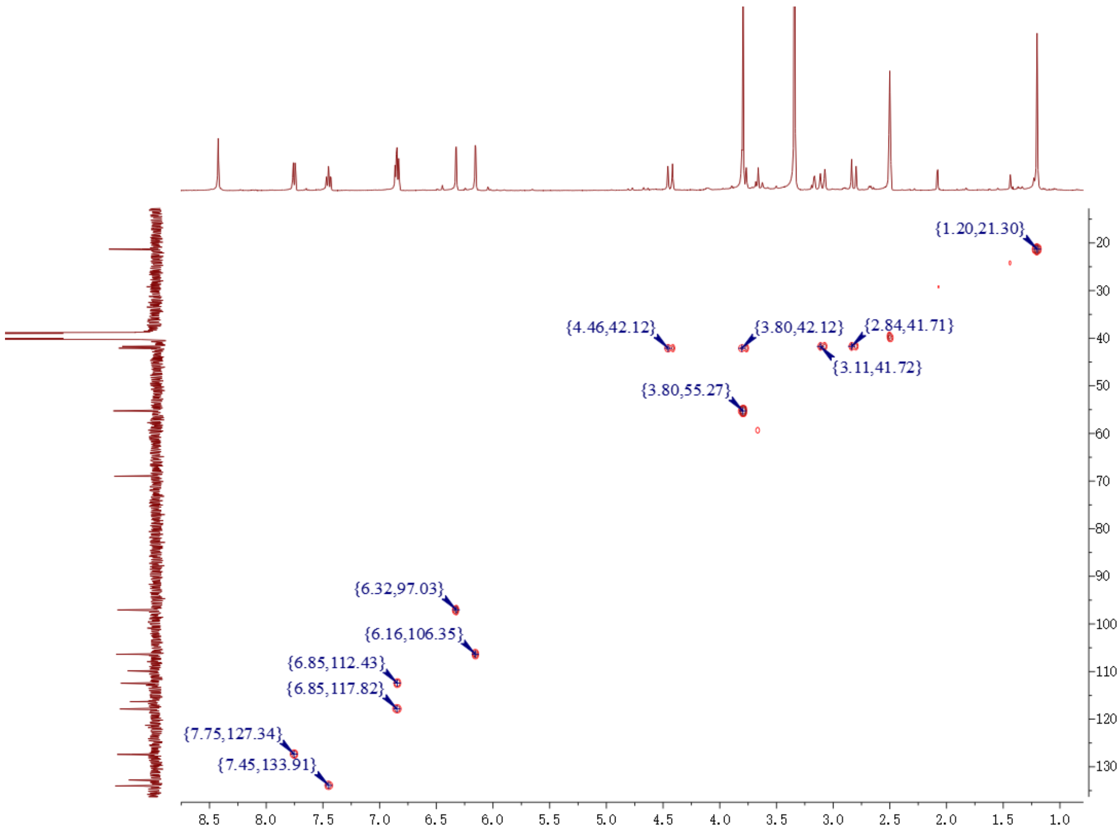

Figure S4. The HSQC spectrum of compound **1** in DMSO-d_6.

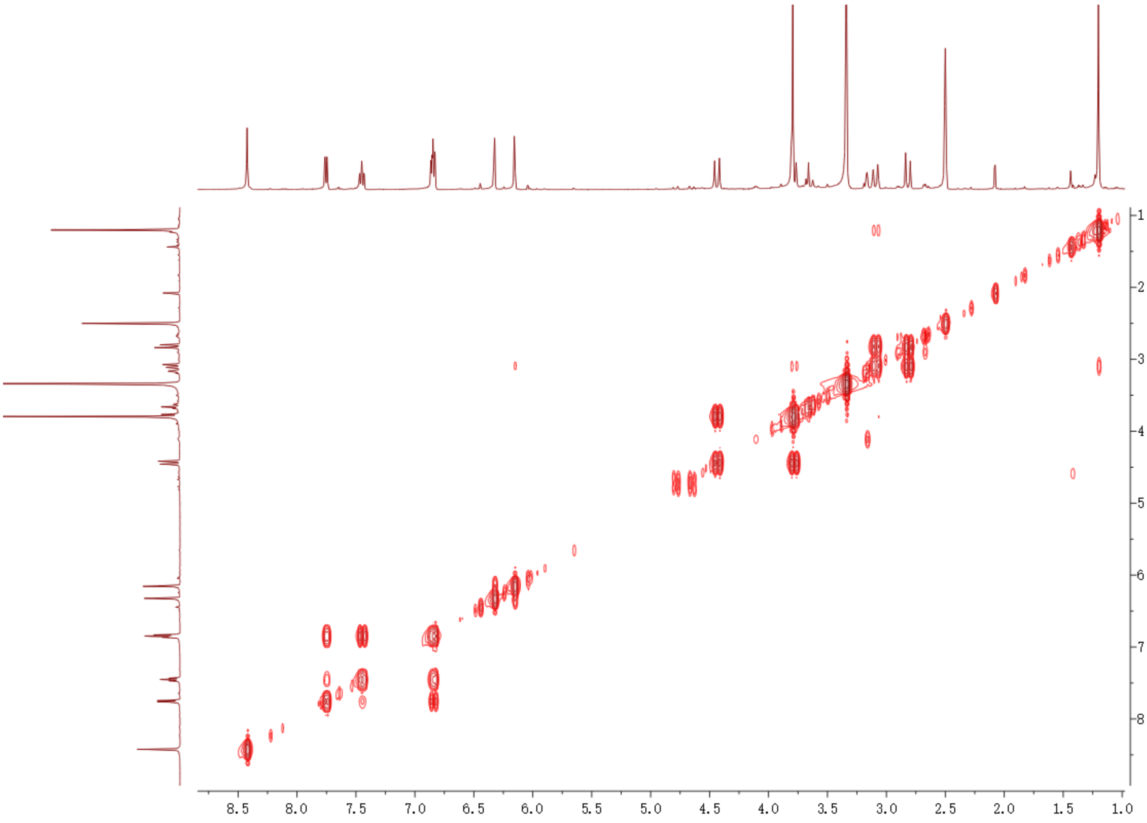

Figure S5. The ^{1}H-^{1}H COSY spectrum of compound **1** in DMSO-d_6.

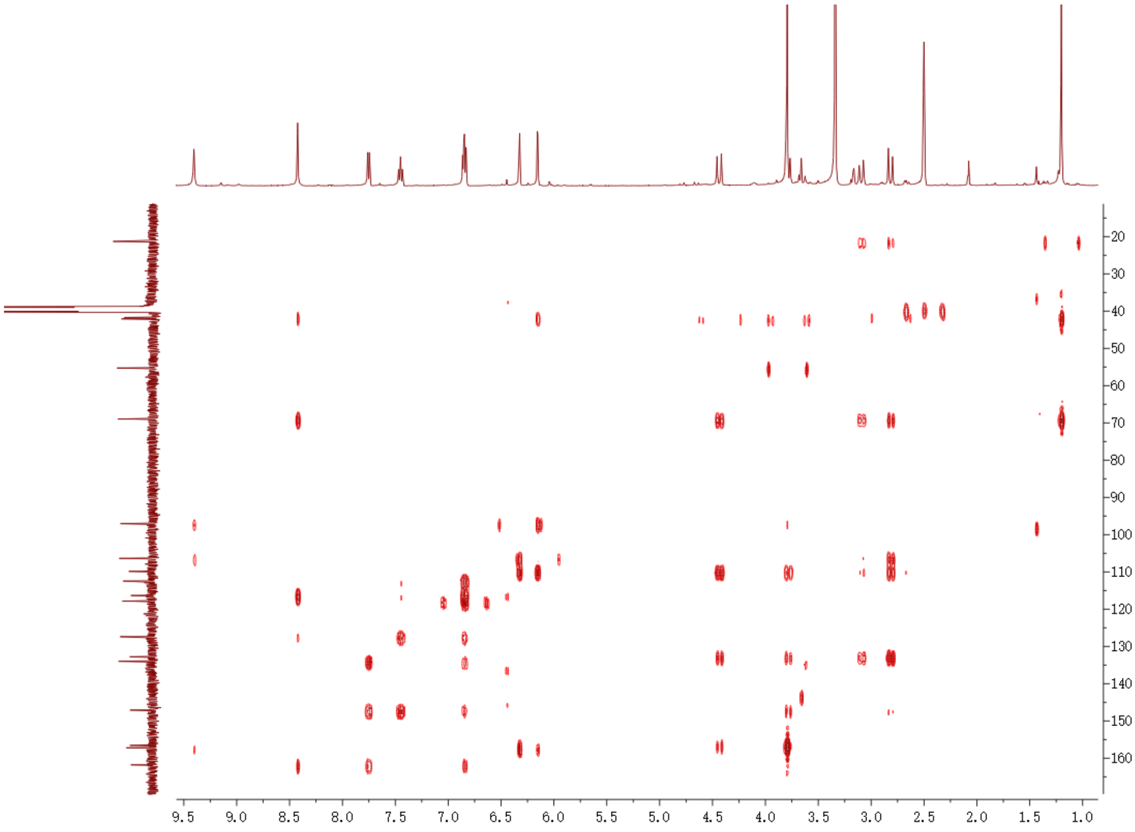

Figure S6. The HMBC spectrum of compound **1** in DMSO-d_6.

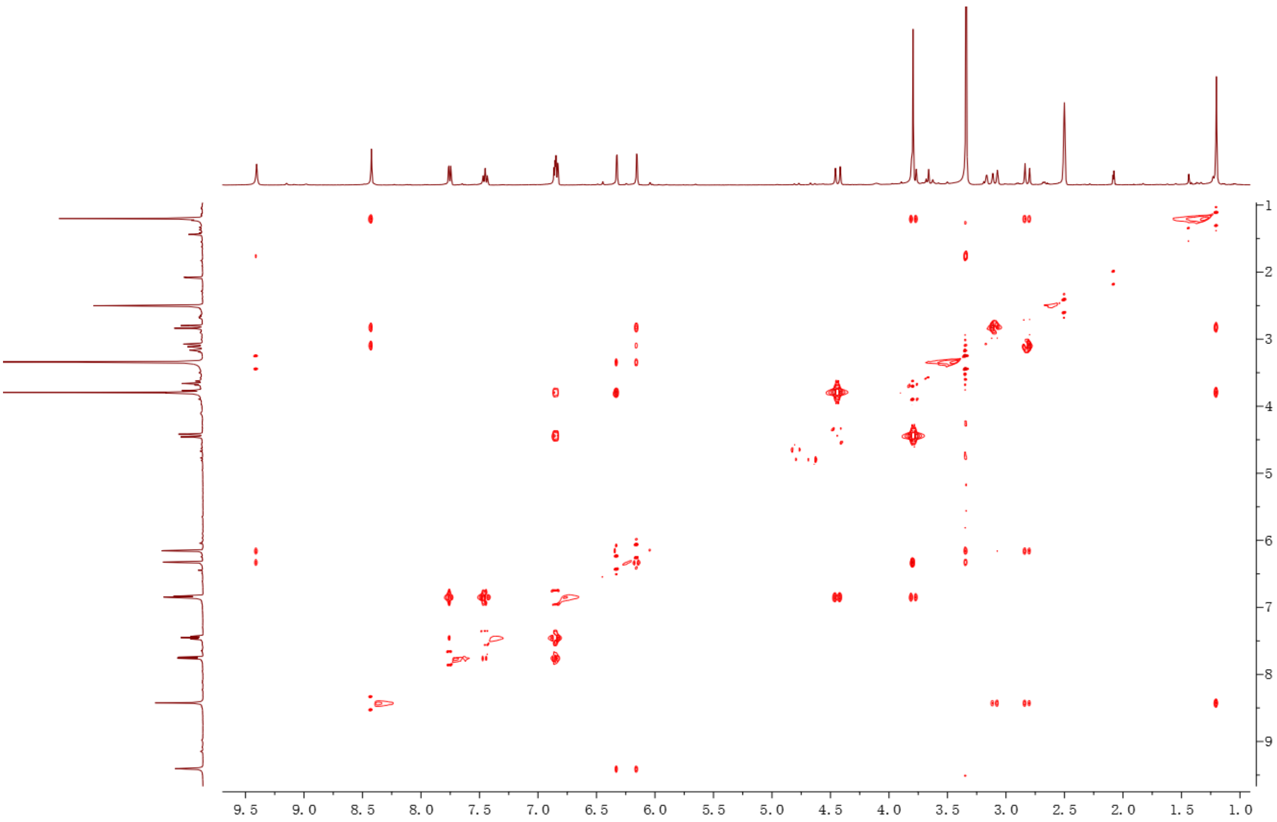

Figure S7. The NOESY spectrum of compound **1** in DMSO-d_6.

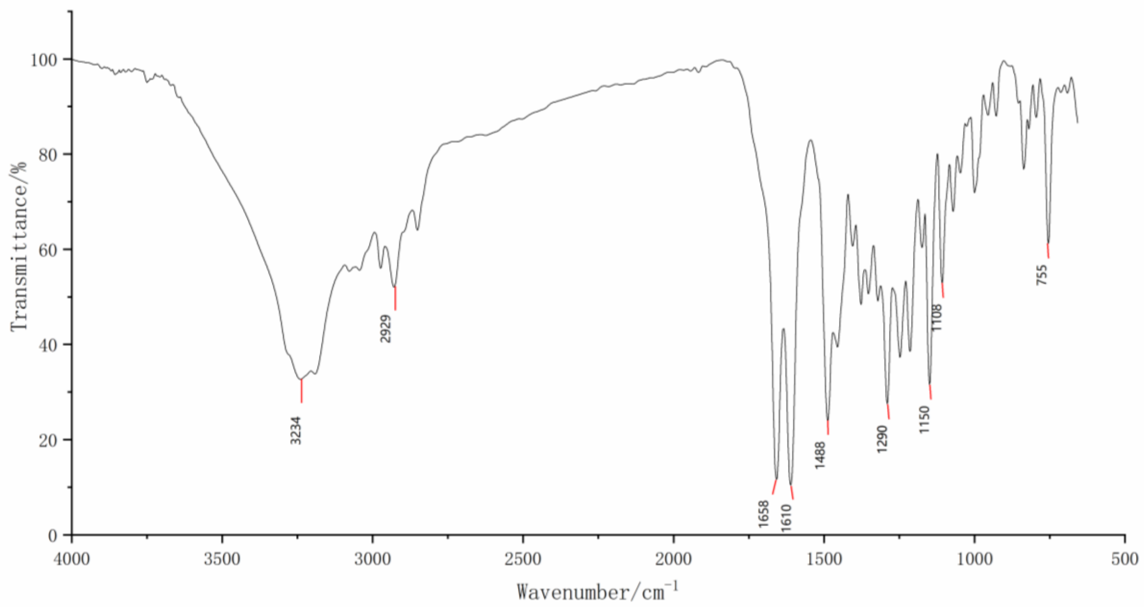

Figure S8. The IR spectrum of compound **1**

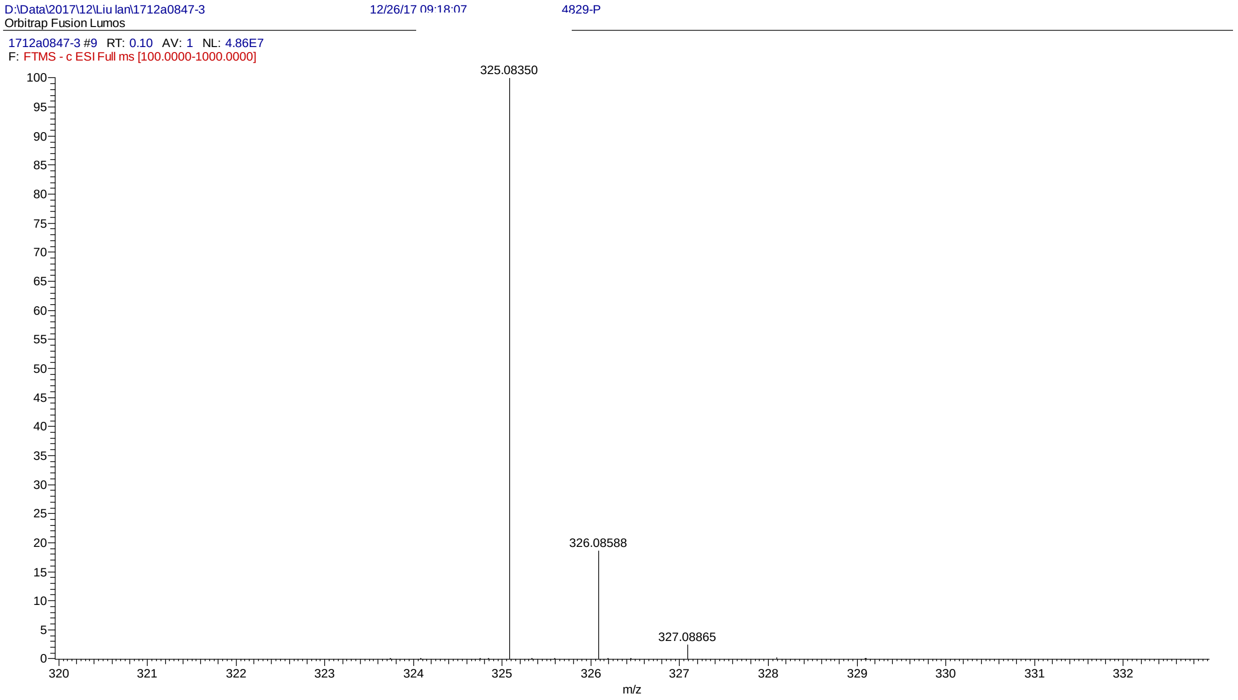

Figure S9. The HRESIMS spectrum of compound **2**.

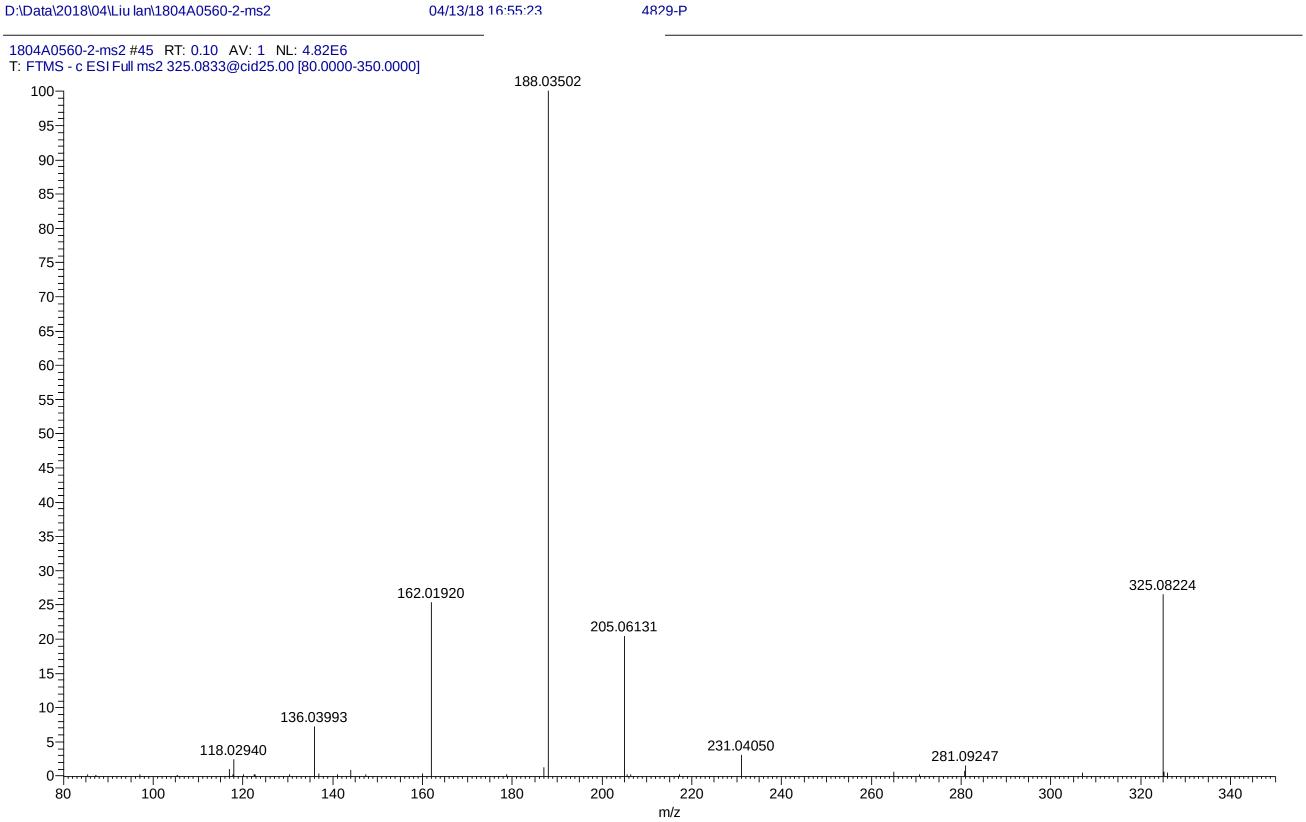

Figure S10. The HR-ESI-MS/MS spectrum of compound **2**.

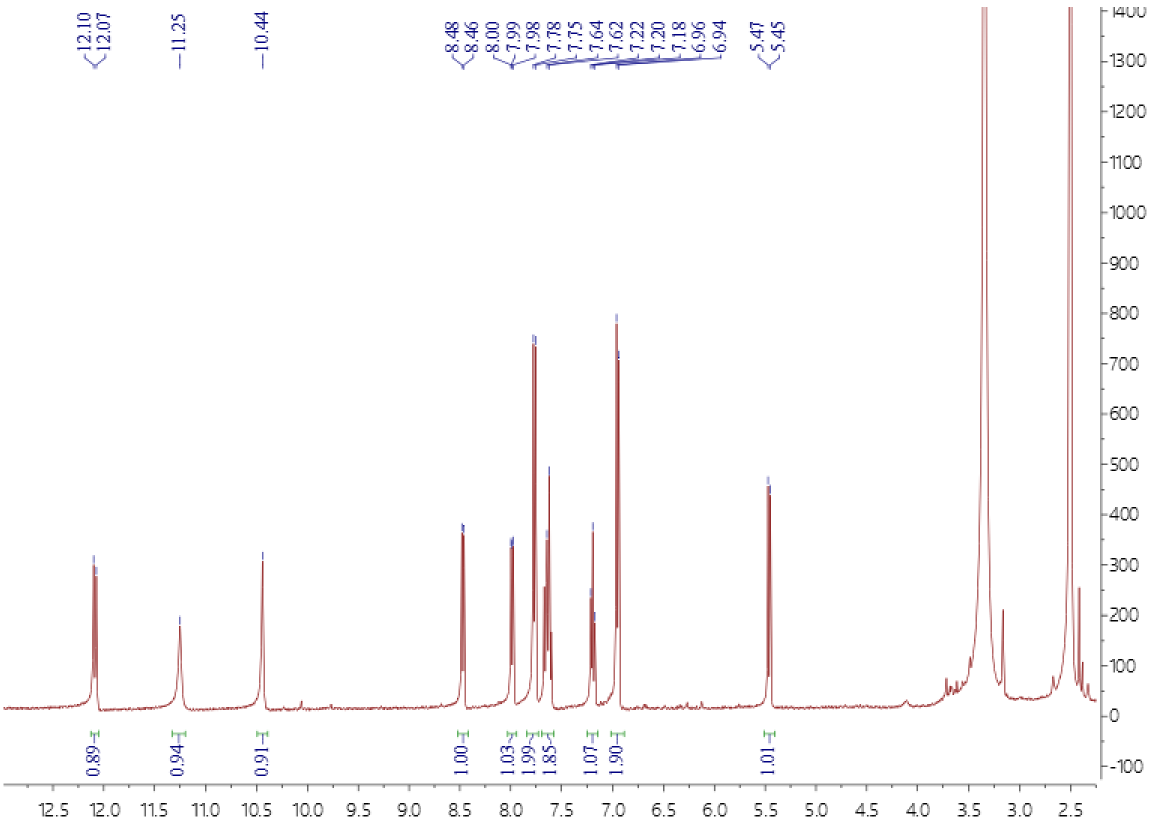

Figure S11. The ^{1}H NMR (400MHz) spectrum of compound **2** in DMSO-d_6.

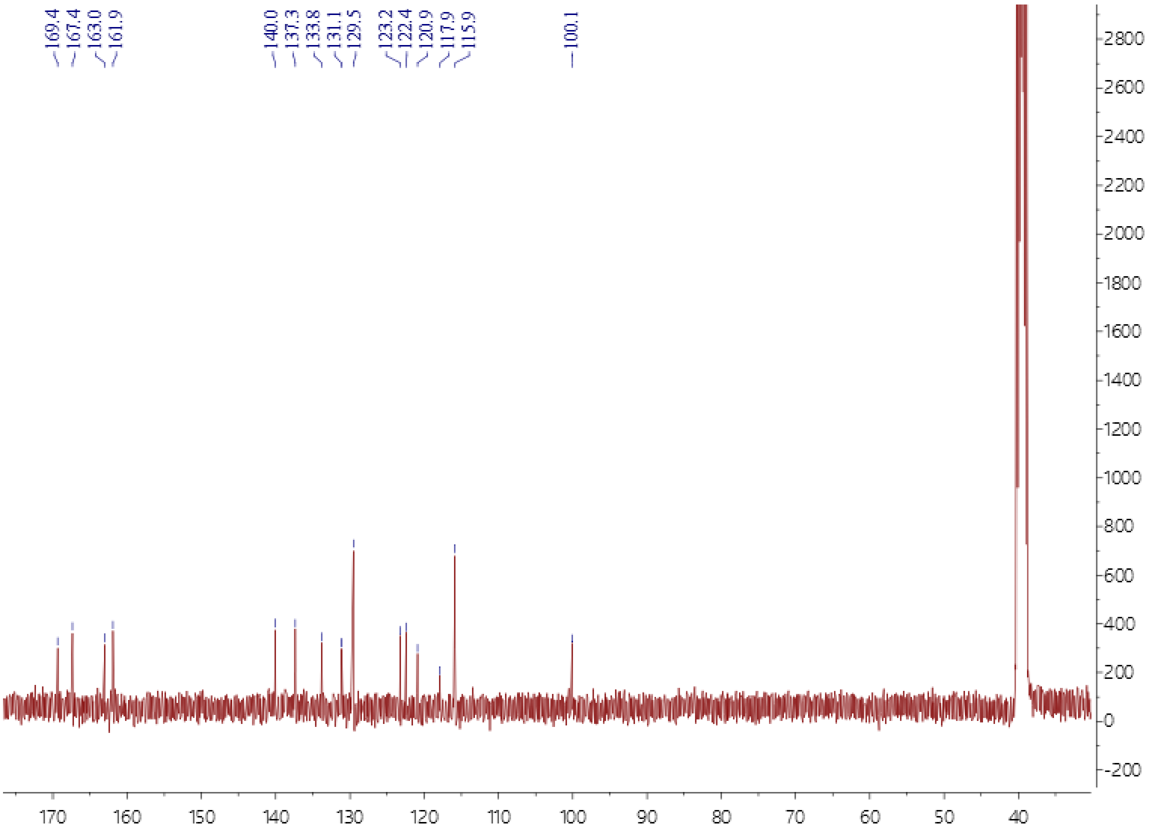

Figure S12. The ^{13}C NMR (100MHz) spectrum of compound **2** in DMSO-d_6.

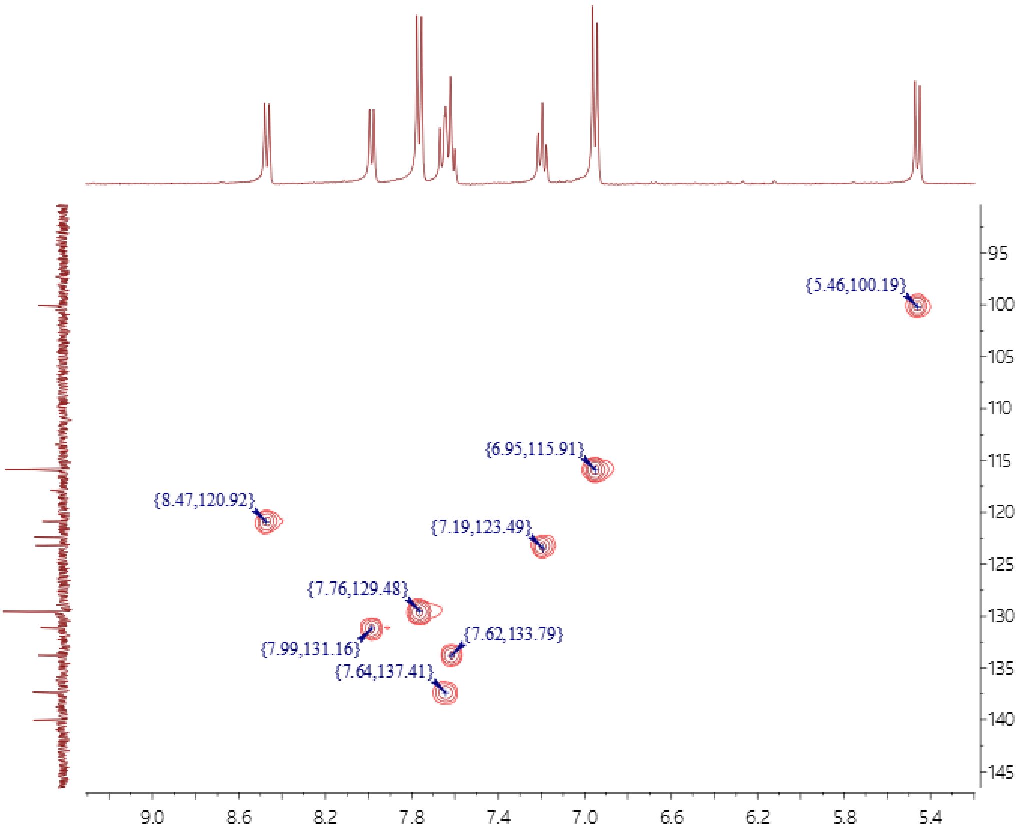

Figure S13. The HSQC spectrum of compound **2** in DMSO-d_6.

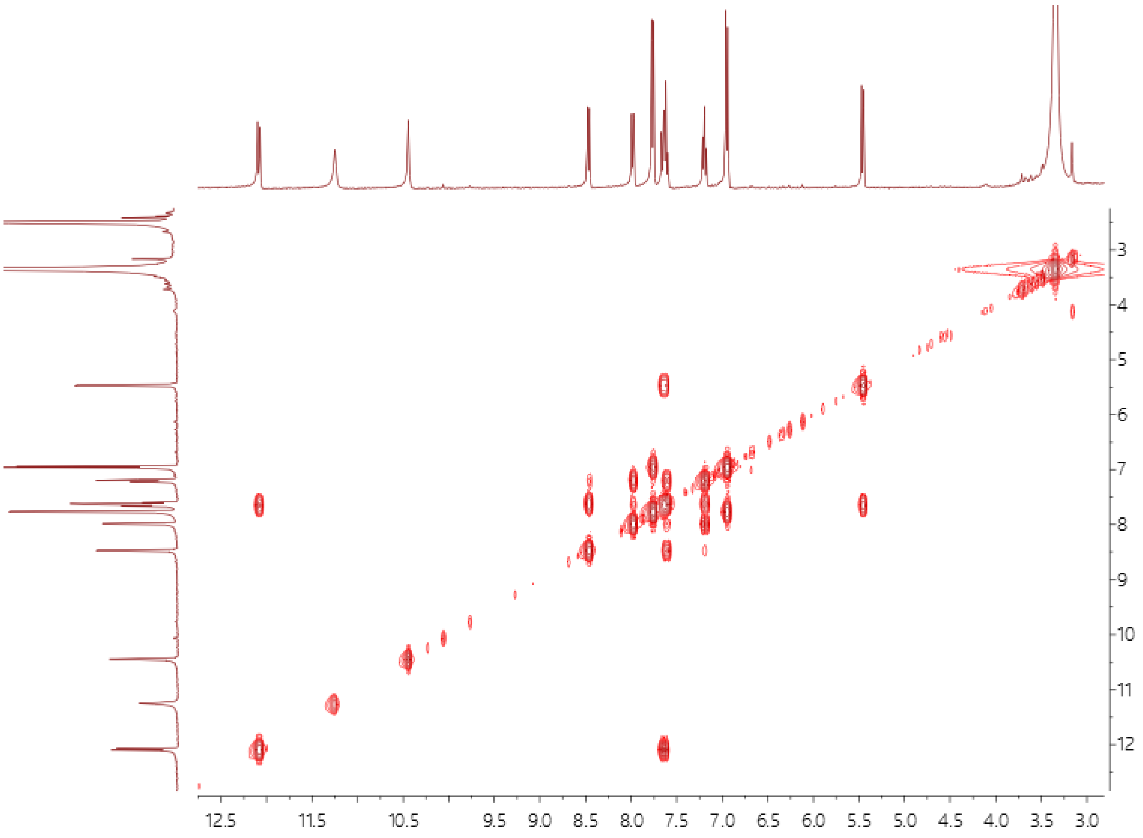

Figure S14. The ^{1}H-^{1}H COSY spectrum of compound **2** in DMSO-d_6.

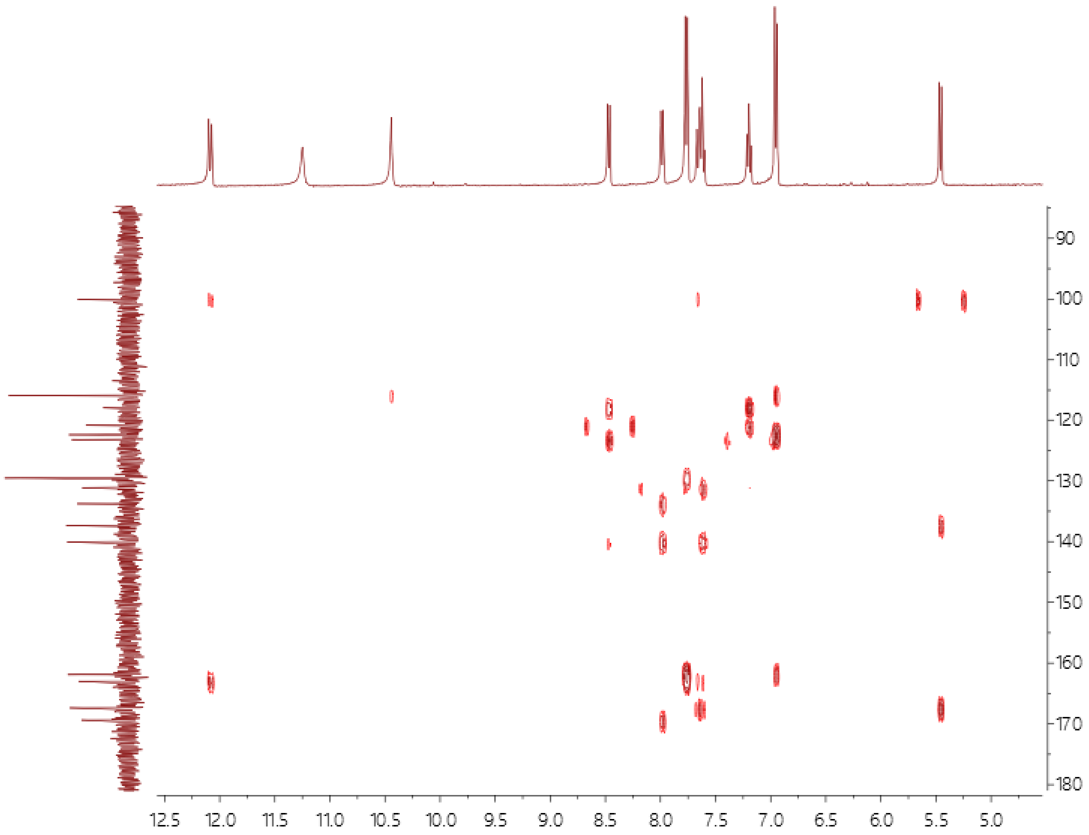

Figure S15. The HMBC spectrum of compound **2** in DMSO-d_6.

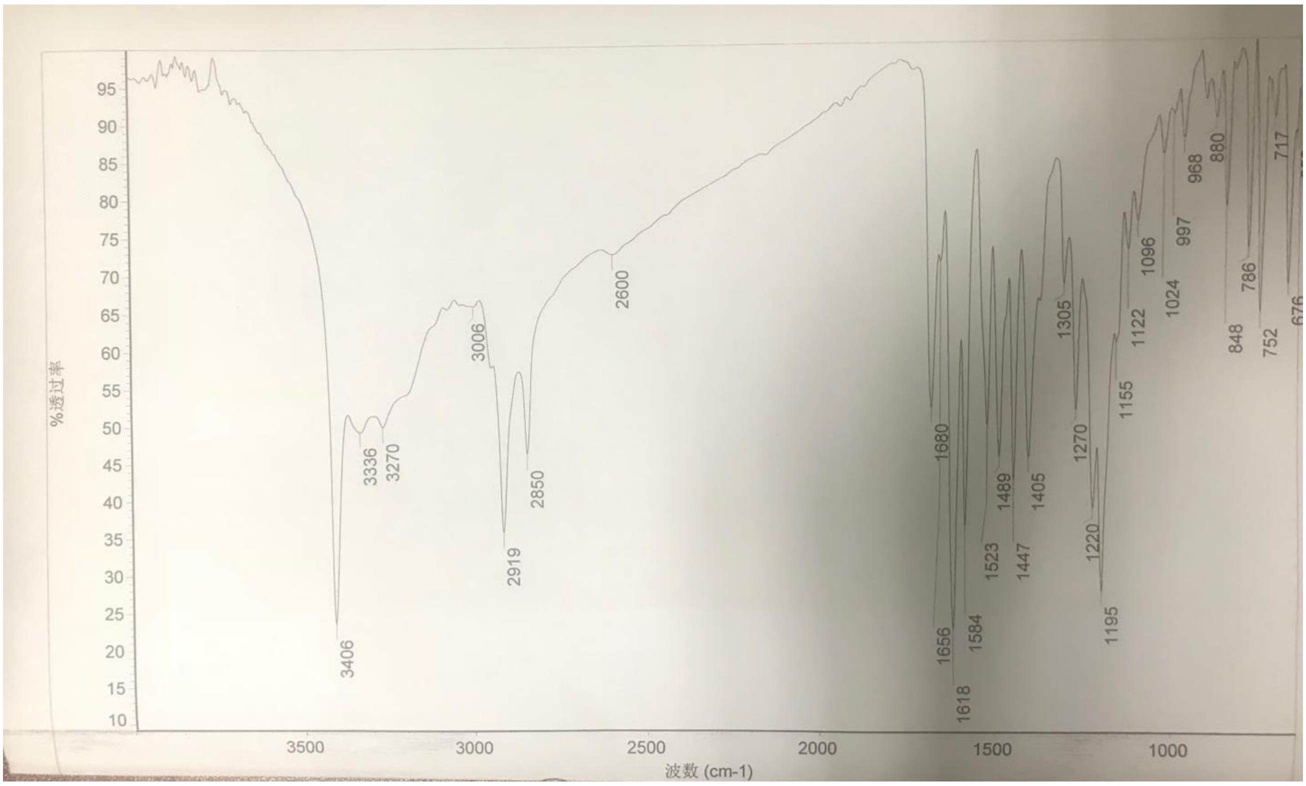

Figure S16. IR spectrum of compound **2**.

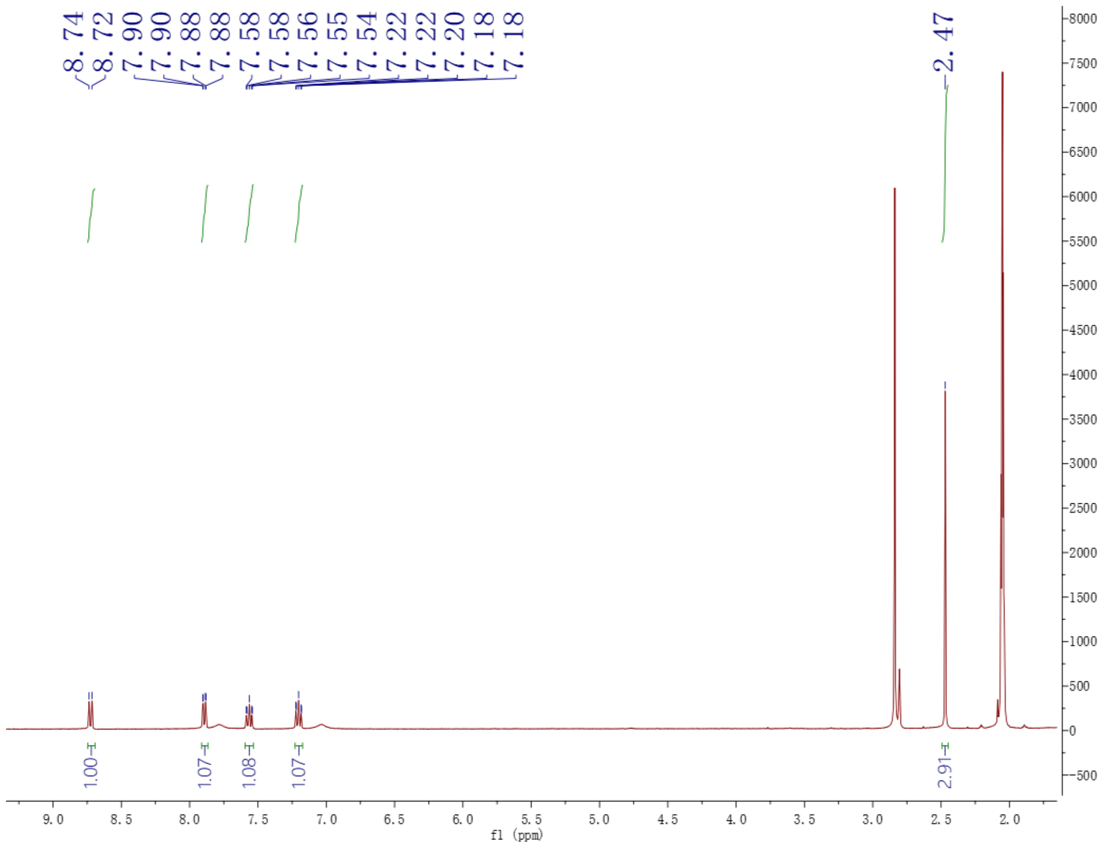

Figure S17. The ^{1}H NMR (400MHz) spectrum of compound **3** in acetone-d_6.

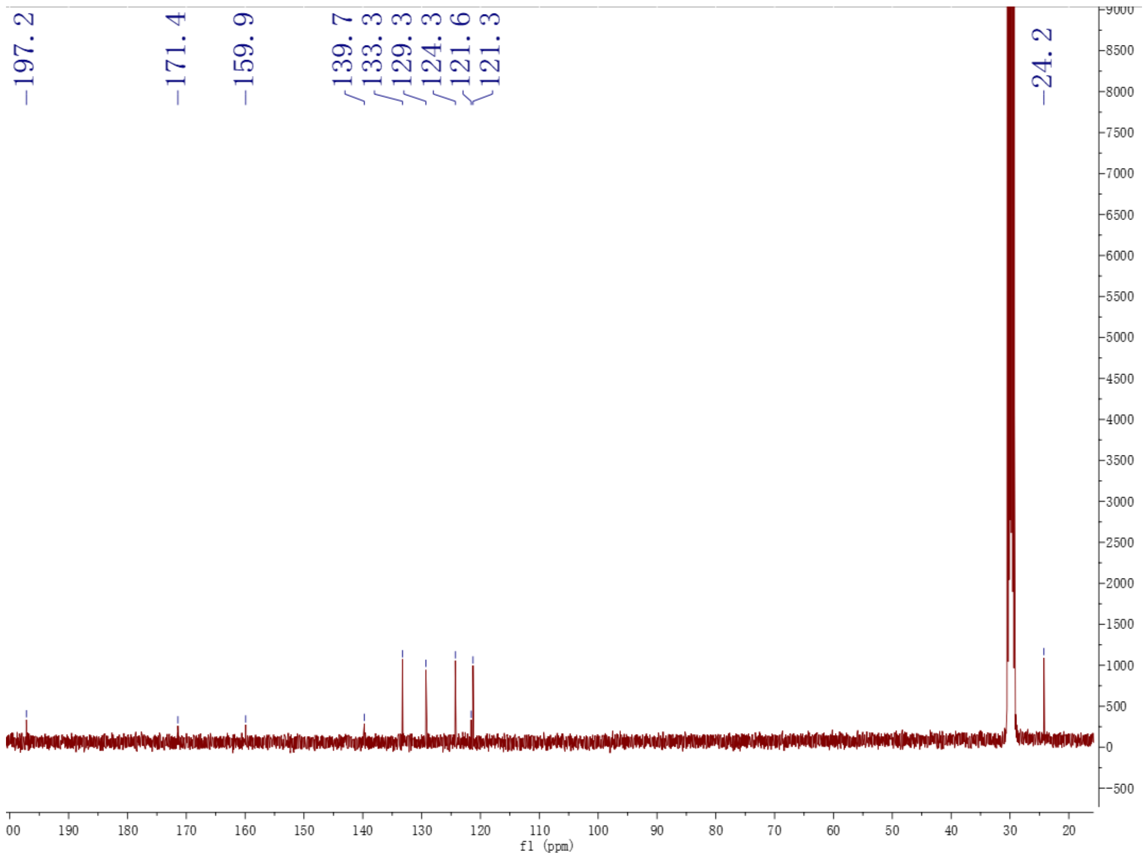

Figure S18. The ^{13}C NMR (100MHz) spectrum of compound **3** in acetone-d_6.

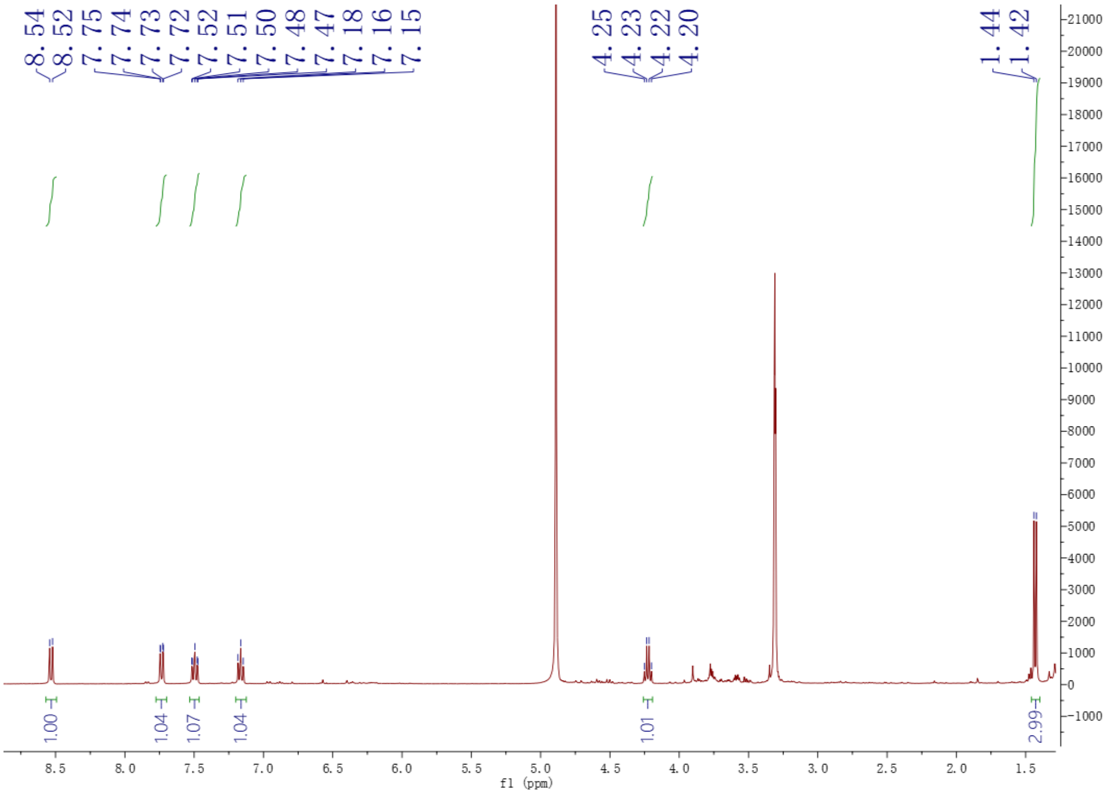

Figure S19. The ^{1}H NMR (400MHz) spectrum of compound **4** in MeOH-d_4.

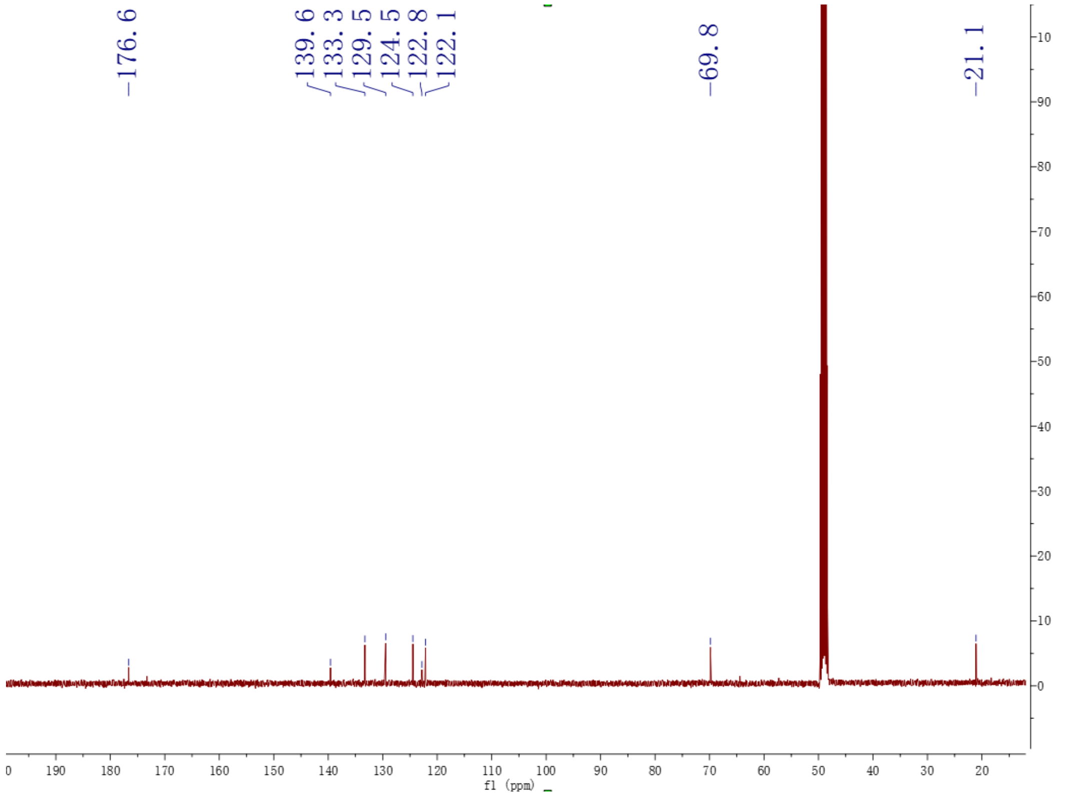

Figure S20. The ^{13}C NMR (100MHz) spectrum of compound **4** in MeOH-d_4.

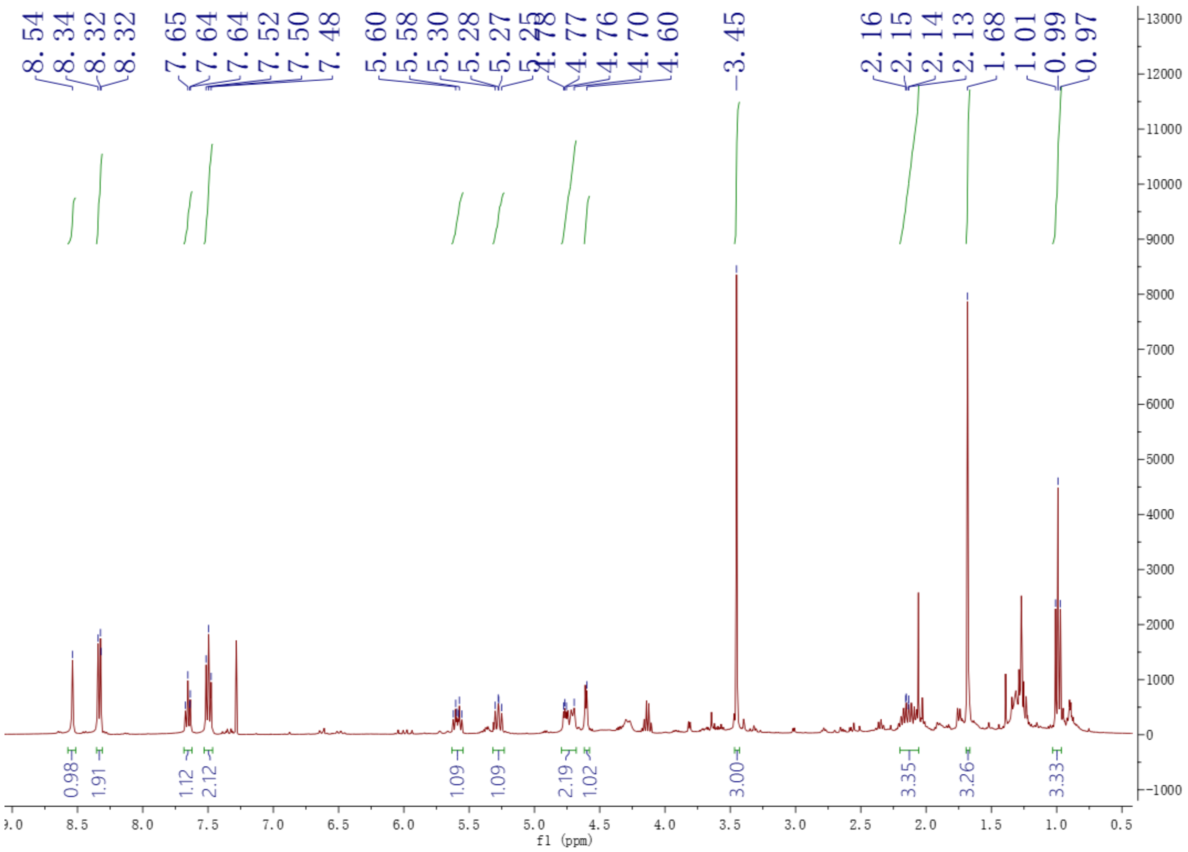

Figure S21. The ^{1}H NMR (400MHz) spectrum of compound **5** in $CDCl_3$.

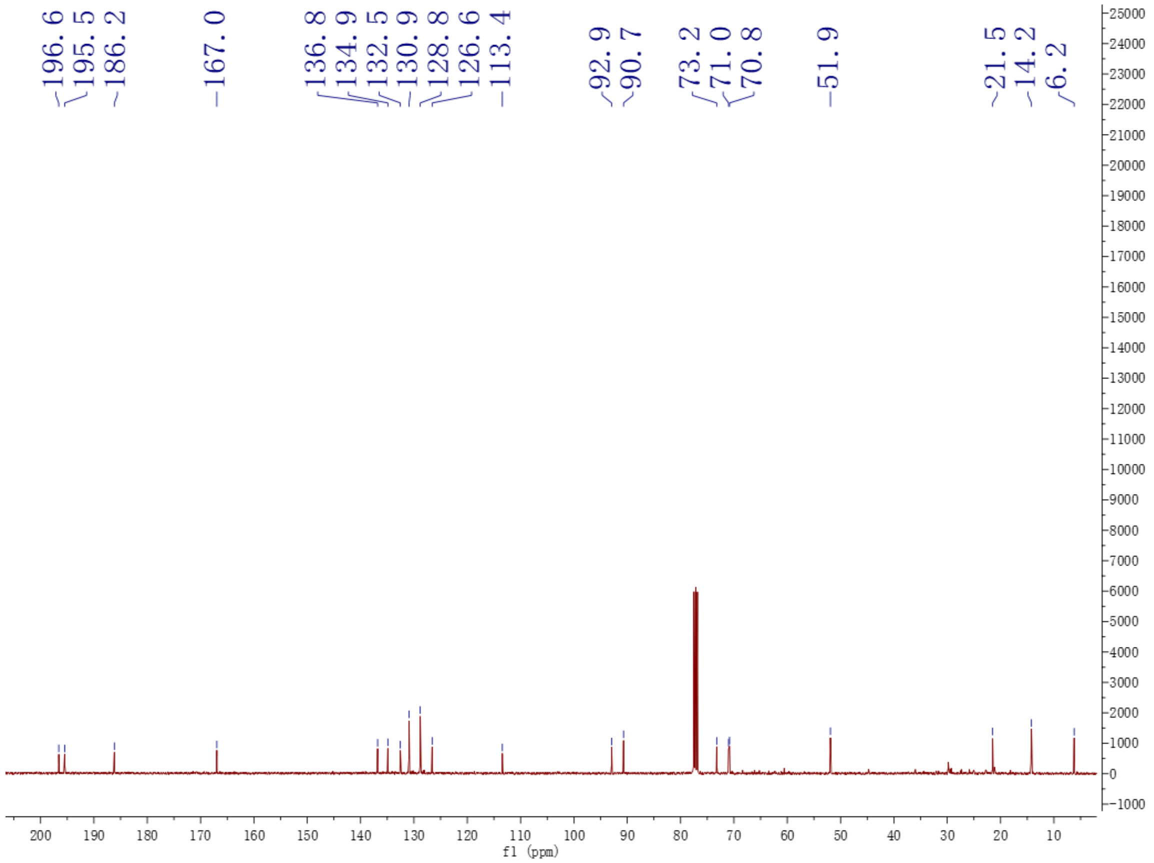

Figure S22. The ^{13}C NMR (100MHz) spectrum of compound **5** in $CDCl_3$.

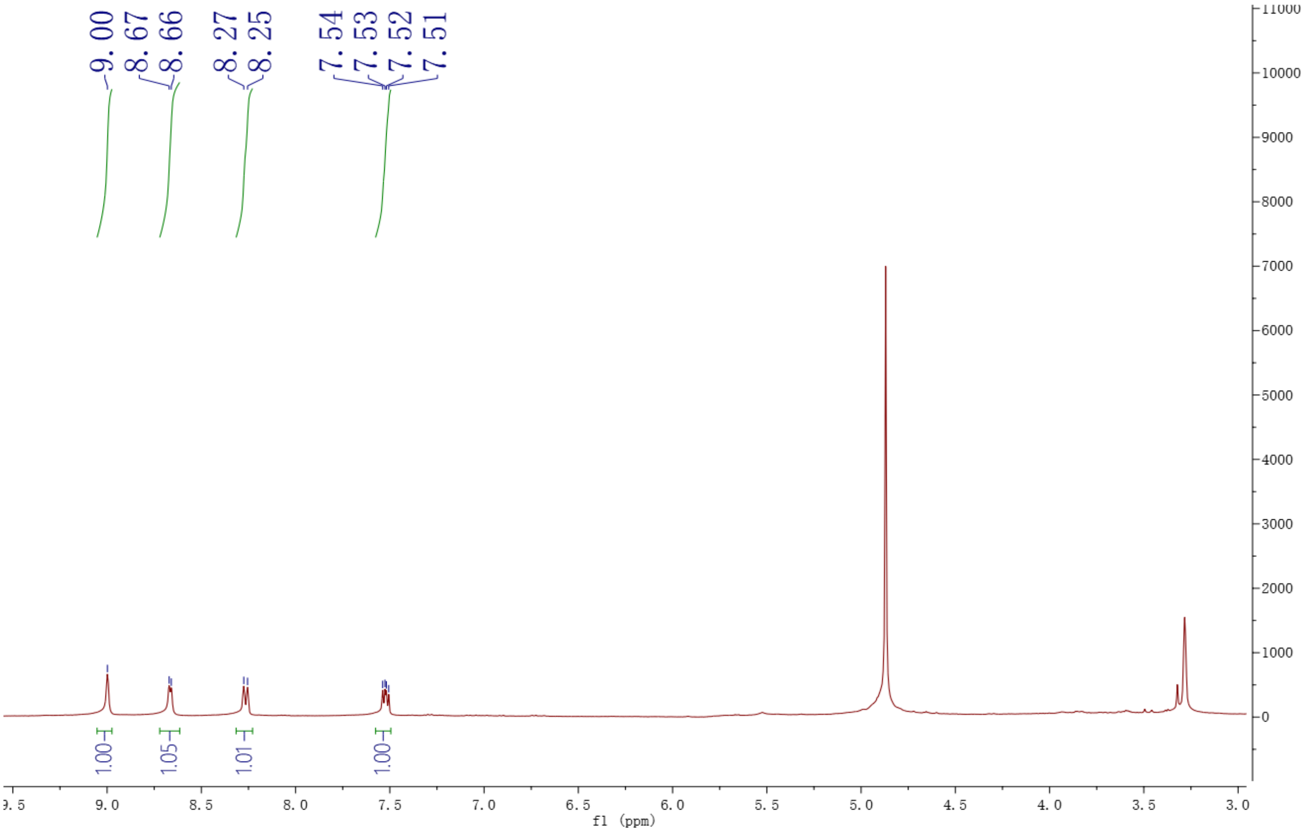

Figure S23. The ^{1}H NMR (400MHz) spectrum of compound **6** in MeOH-d_4.

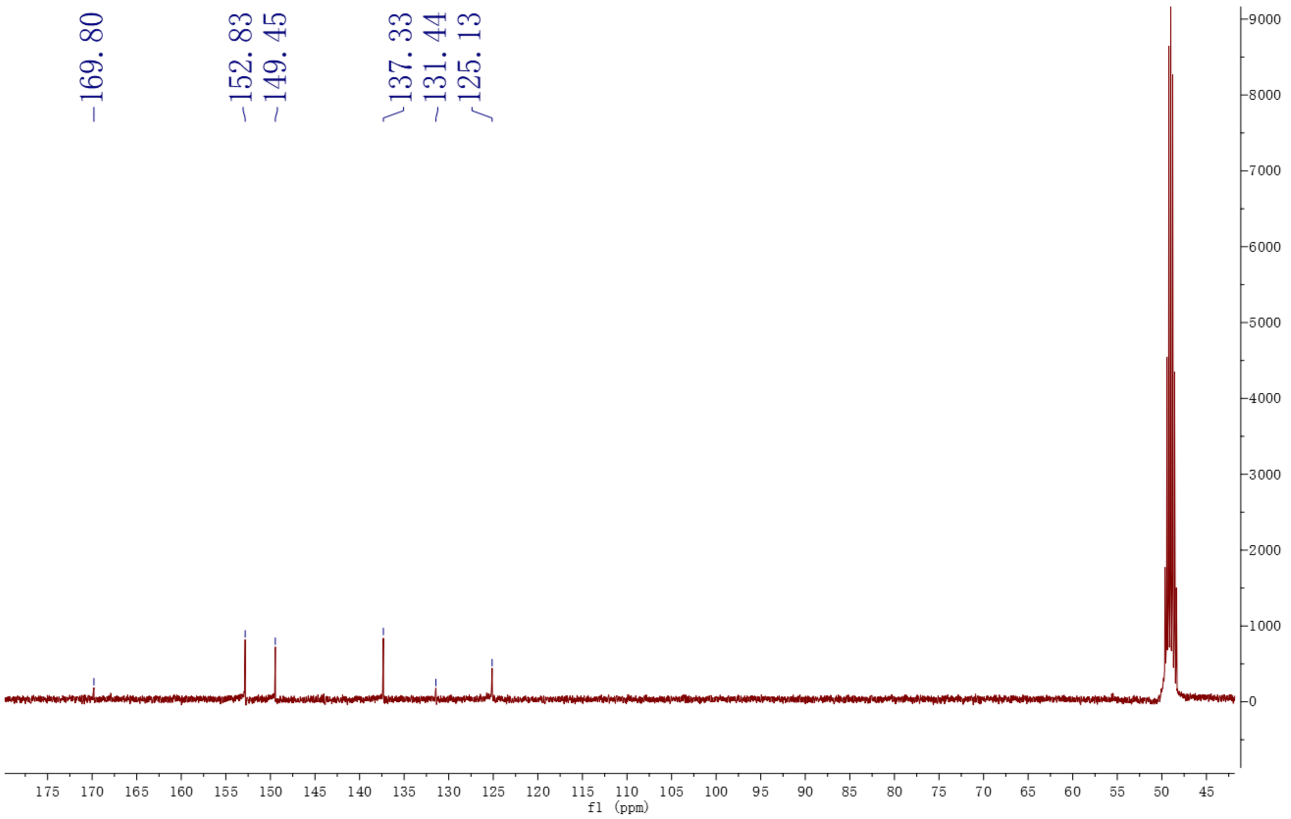

Figure S24. The ^{13}C NMR (100MHz) spectrum of compound **6** in MeOH-d_4.

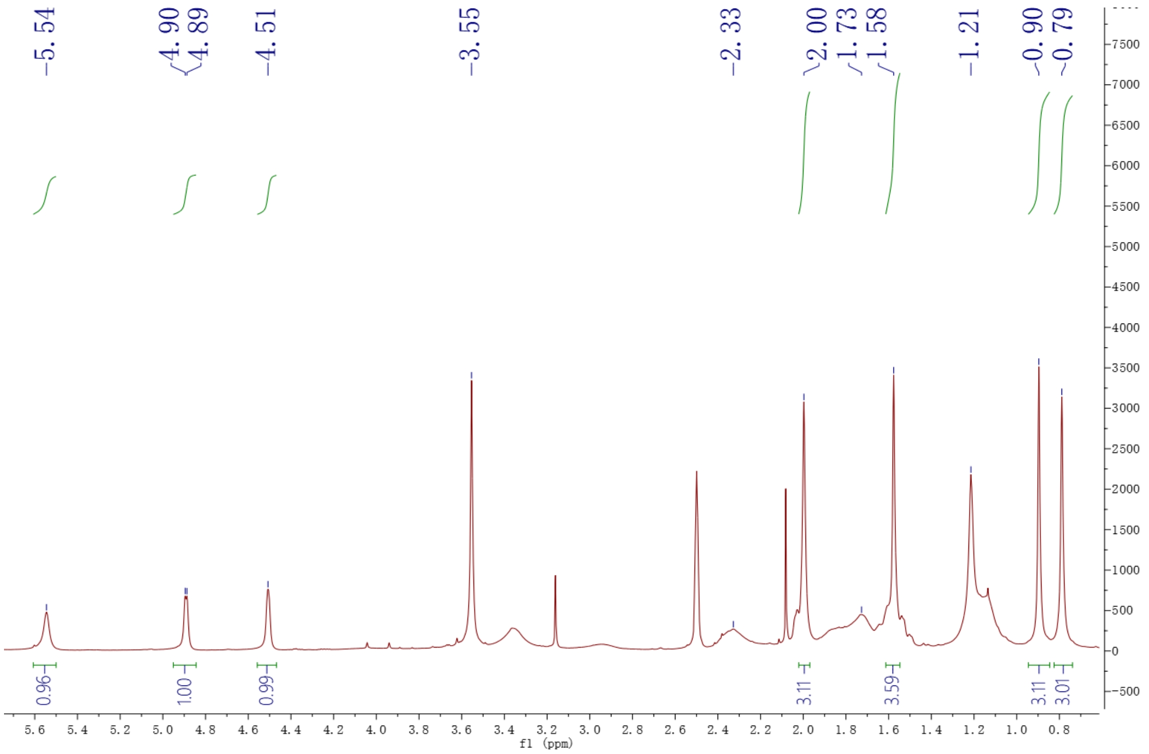

Figure S25. The ^{1}H NMR (400MHz) spectrum of compound **7** in DMSO-d_6.

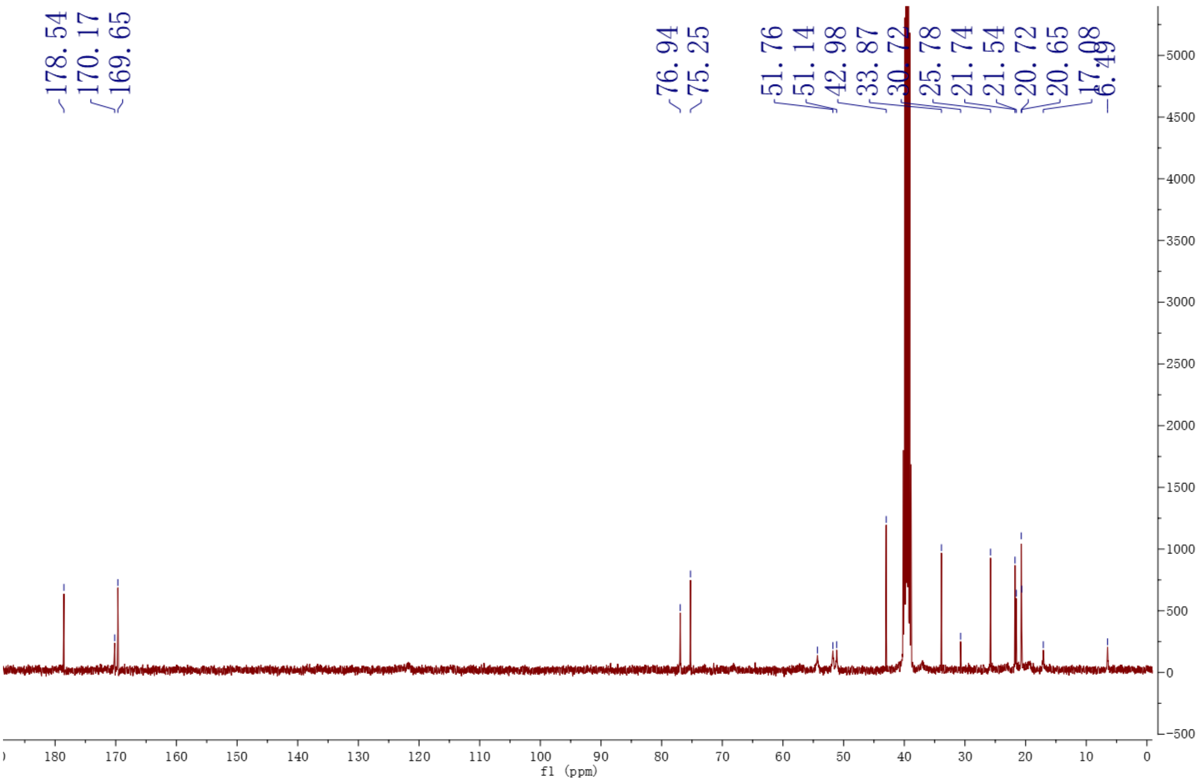

Figure S26. The ^{13}C NMR (100MHz) spectrum of compound **7** in DMSO-d_6.

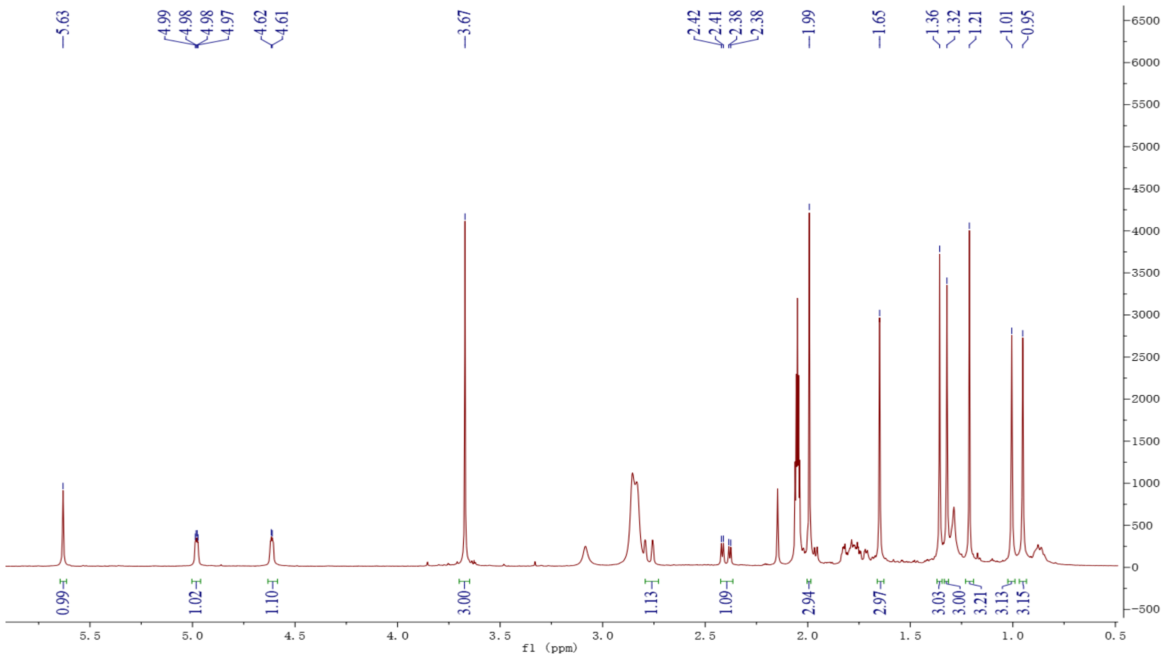

Figure S27. The ^{1}H NMR (400MHz) spectrum of compound **8** in acetone-d_6.

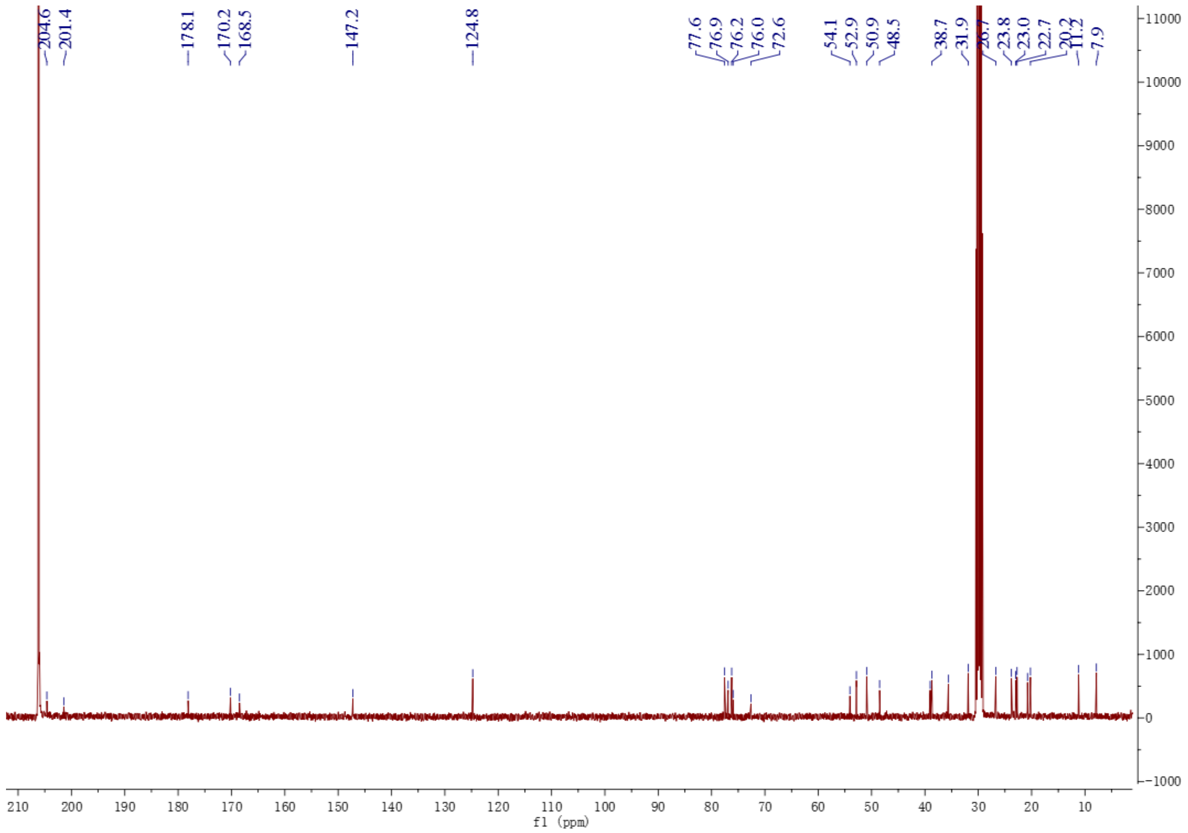

Figure S28. The ^{13}C NMR (100MHz) spectrum of compound **8** in acetone-d_6.

Experimental Section

Calculation of ECD Spectra

Molecular Merck force field (MMFF) and DFT/TD-DFT calculations were carried out with Spartan' 14 software (Wavefunction Inc., Irvine, CA, USA) and Gaussian 09 program, respectively[1]. Conformers within 10 kcal/mol energy window were generated and optimized using DFT calculations at B3LYP/6-31G(d) level. Conformers with Bolzmann distribution over 1% were chosen for ECD calculations in methanol at B3lYP/6-311+g(2d,p) level. The IEF-PCM solvent model for MeOH was used. ECD spectra were generated using the program SpecDis 3.0 (University of Würzburg, Würzburg, Germany) and OriginPro 8.5 (OriginLab, Ltd., Northampton, MA, USA) from dipole-length rotational strengths by applying Gaussian band shapes with sigma = 0.30 ev. All calculations were performed by Tianhe-2 in National Super Computer Center in Guangzhou.

Table S1. Energy Analysis for the Conformers of (*R*)-1.

compound	Conformation	G (Hartree)	G (Kcal/mol)	ΔG (Kcal/mol)	Boltzmann Dist (%)
(*R*)-**1**	**1-1**	−1032.24391394	−647742.8303	0	60.21
(*R*)-**1**	**1-2**	−1032.24352309	−647742.5851	0.245262076	39.79

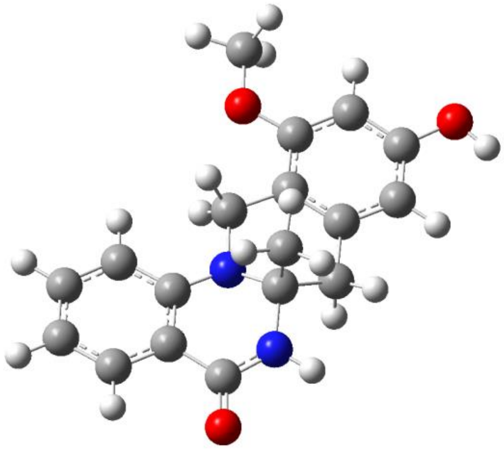

1-1

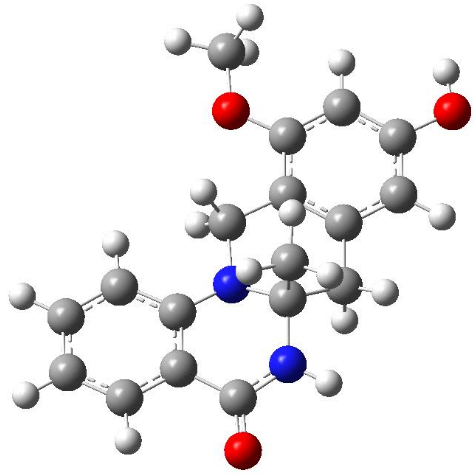

1-2

Figure S17. B3LYP/6-31G(d) optimized low-energy conformers of (*R*)-1.

NMR data of known compounds 3-8.

Compound 3: ^{1}H NMR (acetone-d_6) δ_H 8.73 (1H, d, J = 8.4 Hz), 7.89 (1H, d, J = 7.9 Hz), 7.56 (1H, m), 7.20 (1H, d, J = 7.6 Hz), 2.47 (3H, q, J = 6.9 Hz); ^{13}C NMR (acetone-d_6) δ_C 197.2, 171.4, 159.9, 139.7, 133.3, 129.3, 124.3, 121.6, 121.3, 24.2.

Compound 4: ^{1}H NMR (MeOH-d_4) δ_H 8.53 (1H, d, J = 8.5 Hz), 7.73 (1H, d, J = 7.8 Hz), 7.50 (1H, m), 7.16 (1H, d, J = 7.6 Hz), 4.23 (1H, q, J = 6.9 Hz), 1.43 (3H, q, J = 6.9 Hz); ^{13}C NMR (MeOH-d_4) δ_C 175.2, 138.2, 131.9, 128.1, 123.1, 121.4, 120.7, 68.4, 19.7.

Compound 5: ^{1}H NMR ($CDCl_3$) 8.51 (1H, s), 8.30 (2H, d, J = 8.4 Hz), 7.63 (2H, d, J = 10.6 Hz), 7.47 (2H, d, J = 7.8 Hz), 5.56 (1H, dt, J = 7.3, 10.5 Hz), 5.56 (1H, dt, J = 7.3, 10.5 Hz), 5.26 (1H, m), 4.74 (1H, dd, J = 4.3, 8.7 Hz), 4.68 (1H, d, J = 9.7 Hz), 4.58 (1H, d, J = 4.5 Hz), 3.44 (3H, s), 2.10 (3H, m), 1.66 (3H, s), 0.97 (3H, t, J = 7.5 Hz); ^{13}C NMR ($CDCl_3$) 196.6, 195.5, 186.2, 166.9, 136.8, 134.9, 132.5, 130.9, 128.8, 126.6, 113.4, 92.9, 90.7, 73.2, 71.0, 70.8, 51.9, 21.5, 14.2, 6.2.

Compound 6: ^{1}H NMR (MeOH-d_4) 9.03 (1H, s, H-3), 8.69 (1H, d, J = 4.3 Hz, H-3), 8.29 (1H, d, J = 8.0 Hz, H-6), 7.55 (1H, d, J = 7.9, 5.0 Hz, H-3); ^{13}C NMR (MeOH-d_4) 169.8, 152.8, 149.4, 137.3, 131.4, 125.1.

Compound 7: ^{1}H NMR (DMSO-d_6) δ_H 5.55 (1H, s), 4.49 (1H, d, J = 3.2 Hz), 4.51 (1H, s), 3.56 (3H, s), 2.32 (2H, m), 2.00 (3H, s), 1.77 (4H, m), 1.58 (3H, s), 1.22 (3H, s), 1.16 (3H, s), 0.90 (3H, s), 0.80 (3H, s); ^{13}C NMR (DMSO-d_6) δ_C 178.5, 170.2, 169.6, 121.8, 114.8, 76.9, 75.2, 54.3, 51.8, 51.1, 43.0, 33.9, 30.7, 25.8, 21.7, 21.5, 20.7, 20.6, 17.1, 6.5.

Compound 8: ^{1}H NMR (acetone-d_6) δ_C 5.63 (1H, s), 4.98 (1H, dd, J = 3.6, 1.6 Hz), 4.61 (1H, dd, J = 3.7, 1.5 Hz), 3.67 (3H, s), 2.78 (1H, br d, J =14.4 Hz), 2.40 (1H, dd, J =14.4, 3.8 Hz), 2.15 (1H, s), 1.98 (2H, m), 1.99 (3H, s), 1.78 (2H, m), 1.65 (3H, s), 1.36 (3H, s), 1.32 (3H, s), 1.21 (3H, s), 1.01 (3H, s), 0.95 (3H, s); ^{13}C NMR (acetone-d_6) δ_C 204.6 (C-15), 201.4 (C-17), 178.1 (C-23), 170.2 (C_3–OAc), 168.5 (C-19), 147.2 (C-9), 124.7 (C-11), 77.6 (C-6), 76.9 (C-12), 76.2 (C-3), 76.0 (C-16), 72.6 (C-14), 54.1 (C-13), 52.5 (C_{19}–OMe), 50.9 (C-5), 48.5 (C-10), 39.0 (C-8), 38.7 (C-7), 35.6 (C-4), 31.9 (C-22), 26.7 (C-24), 23.8 (C-21), 23.0 (C-25), 22.7 (C-2), 20.8 (C_3–OAc), 20.2 (C-1), 11.2 (C-20), 7.8 (C-18).

References.

1. Frisch, M. J.; Trucks, G. W.; Schlegel, H. B.; Scuseria, G. E.; Robb, M. A.; Cheeseman, J. R.; Scalmani, G.; Barone, V.; Mennucci, B.; Petersson, G. A.; Nakatsuji, H.; Caricato, M.; Li, X.; Hratchian, H. P.; Izmaylov, A. F.; Bloino, J.; Zheng, G.; Sonnenberg, J. L.; Hada, M.; Ehara, M.; Toyota, K.; Fukuda, R.; Hasegawa, J.; Ishida, M.; Nakajima, T.; Honda, Y.; Kitao, O.; Nakai, H.; Vreven, T.; Montgomery, J. A.; Peralta, J. E.; Ogliaro, F.; Bearpark, M.; Heyd, J. J.; Brothers, E.; Kudin, K. N.; Staroverov, V. N.; Keith, T.; Kobayashi, R.; Normand, J.; Raghavachari, K.; Rendell, A.; Burant, J. C.; Iyengar, S. S.; Tomasi, J.; Cossi, M.; Rega, N.; Millam, J. M.; Klene, M.; Knox, J. E.; Cross, J. B.; Bakken, V.; Adamo, C.; Jaramillo, J.; Gomperts, R.; Stratmann, R. E.; Yazyev, O.; Austin, A. J.; Cammi, R.; Pomelli, C.; Ochterski, J. W.; Martin, R. L.; Morokuma, K.; Zakrzewski, V. G.; Voth, G. A.; Salvador, P.; Dannenberg, J. J.; Dapprich, S.; Daniels, A. D.; Farkas, O.; Foresman, J. B.; Ortiz, J. V.; Cioslowski, J.; Fox, D. J. Gaussian 09, revision C.01. Gaussian, Inc.: Wallingford CT, 2010.Bruhn, T.; Schauml öffel, A.; Hemberger, Y.; Bringmann, G. SpecDis: Quantifying the comparison of calculated and experimental electronic circular dichroism spectra. Chirality 2013, 25, 243–249.
